# *In vivo* regulation of glycogen synthase kinase 3β activity in neurons and brains

**DOI:** 10.1038/s41598-017-09239-5

**Published:** 2017-08-17

**Authors:** Ambika Krishnankutty, Taeko Kimura, Taro Saito, Kyota Aoyagi, Akiko Asada, Shin-Ichiro Takahashi, Kanae Ando, Mica Ohara-Imaizumi, Koichi Ishiguro, Shin-ichi Hisanaga

**Affiliations:** 1 Laboratory of Molecular Neuroscience, Department of Biological Sciences, Graduate School of Science, Tokyo Metropolitan University, Hachioji, Tokyo Japan; 2 Department of Biochemistry, Kyorin University School of Medicine, Mitaka, Tokyo Japan; 3 Department of Animal Sciences, Graduate School of Agriculture and Life Sciences, The University of Tokyo, Bunkyo, Tokyo Japan; 4 Department of Neurology, Graduate School of Medicine, Juntendo University, Bunkyo, Tokyo Japan

## Abstract

Glycogen synthase kinase 3β (GSK3β) is a multifunctional protein kinase involved in many cellular activities including development, differentiation and diseases. GSK3β is thought to be constitutively activated by autophosphorylation at Tyr216 and inactivated by phosphorylation at Ser9. The GSK3β activity has previously been evaluated by inhibitory Ser9 phosphorylation, but it does not necessarily indicate the kinase activity itself. Here, we applied the Phos-tag SDS-PAGE technique to the analysis of GSK3β phosphoisotypes in cells and brains. There were three phosphoisotypes of GSK3β; double phosphorylation at Ser9 and Tyr216, single phosphorylation at Tyr216 and the nonphosphorylated isotype. Active GSK3β with phosphorylation at Tyr216 represented half or more of the total GSK3β in cultured cells. Although levels of phospho-Ser9 were increased by insulin treatment, Ser9 phosphorylation occurred only in a minor fraction of GSK3β. In mouse brains, GSK3β was principally in the active form with little Ser9 phosphorylation, and the phosphoisotypes of GSK3β changed depending on the regions of the brain, age, sex and disease conditions. These results indicate that the Phos-tag SDS-PAGE method provides a simple and appropriate measurement of active GSK3β *in vivo*, and the activity is regulated by the mechanism other than phosphorylation on Ser9.

## Introduction

Glycogen synthase kinase 3β (GSK3β) is a multifunctional protein kinase that targets Ser/Thr residues with a priming phosphorylation at the fourth amino acid on the C-terminal side^1, 2^. GSK3β is ubiquitously expressed in all types of cells and tissues and is particularly abundant in the brain. GSK3β regulates many cellular functions, including cell proliferation, cell survival, gene expression, cellular architecture, neural development and plasticity etc.^3, 4^. In addition to these physiological functions, GSK3β is also involved in cancers, diabetes and neurodegenerative diseases. In Alzheimer’s disease (AD), GSK3β produces pathological and abnormal phosphorylation of tau^5–7^, but it is not known how GSK3β becomes to phosphorylate tau abnormally in AD brains. It is important to understand the mechanisms that regulate the kinase activity of GSK3β to clarify its versatile functions.

The regulation of GSK3β activity is well studied *in vitro* ^2–4^. GSK3β requires phosphorylation at Tyr216 in the activation loop for activation^8, 9^. Although GSK3β is a Ser/Thr kinase, this Tyr phosphorylation is thought to be autophosphorylation^10^; however, phosphorylation by Src family tyrosine kinases has also been reported^11^. Tyr216-phosphorylated GSK3β is constitutively active and is inactivated by phosphorylation at the N-terminal Ser9 by several protein kinases, such as PKA, PKB, p90RSK and Akt^2–4, 12, 13^. Phosphorylated Ser9 binds to a pocket for a priming phosphorylation in the substrate-binding region and to reduce the binding affinity for substrates^14, 15^.

However, the cellular mechanisms that regulate GSK3β are not yet fully understood. For example, it is not known what amount of GSK3β remains inactive without Tyr216 phosphorylation. Therefore, it is unclear whether all GSK3β molecules are automatically activated by autophosphorylation after *de novo* synthesis or if they require any signal for activation. On the other hand, many signaling pathways are reported to downregulate GSK3β activity by phosphorylation at Ser9^2–4, 12, 13^. For instance, insulin and other growth factors activate Akt, which in turn phosphorylates GSK3β at Ser9, rendering the kinase inactive and resulting in decreased phosphorylation of downstream substrates, such as glycogen synthase, tau, β-catenin, etc.^2–7, 16^. Although some reports measured reduced GSK3β activity in cultured cells upon stimulation with growth factors^17^, no simple method is available to estimate the amount of active GSK3β *in vivo*.

Because it was shown that phosphorylation at Ser9 inhibits GSK3β activity, most previous studies have used the increase or decrease in the phospho-Ser9 levels as changes of cellular GSK3β activity^2–4, 12, 13^. However, it is hard to correctly estimate the kinase activity using the levels of inhibitory phosphorylation. The immunoblotting data showing an increase in the phospho-Ser9 levels in cells after growth factor treatment do not provide the absolute extent of inactivation. For example, a 2-fold increase in the phospho-Ser9 levels did not discriminate the 1 to 2% or 50 to 100% changes in the total GSK3β levels. We thought that it would be much more informative to measure the active form of GSK3β if possible. We have previously used Phos-tag SDS-PAGE, which was first reported by Kinoshita *et al*.^18^, to reveal the *in vivo* phosphorylation states of p35 Cdk5 activator, and indicated the usefulness of the method in quantitatively measuring the phosphoisotypes of proteins^19^. In this study, we measured the absolute amount of active GSK3β in cultured cells, primary neurons and mouse brains using the Phos-tag technique. In fact, we could measure the active form of GSK3β *in vivo* and found that the amount of active GSK3β changed in brains depending on the regions, ages, sex and disease conditions.

## Results

### Identification of the phosphorylation states of GSK3β in cells using Phos-tag SDS-PAGE

**Figure 1:**
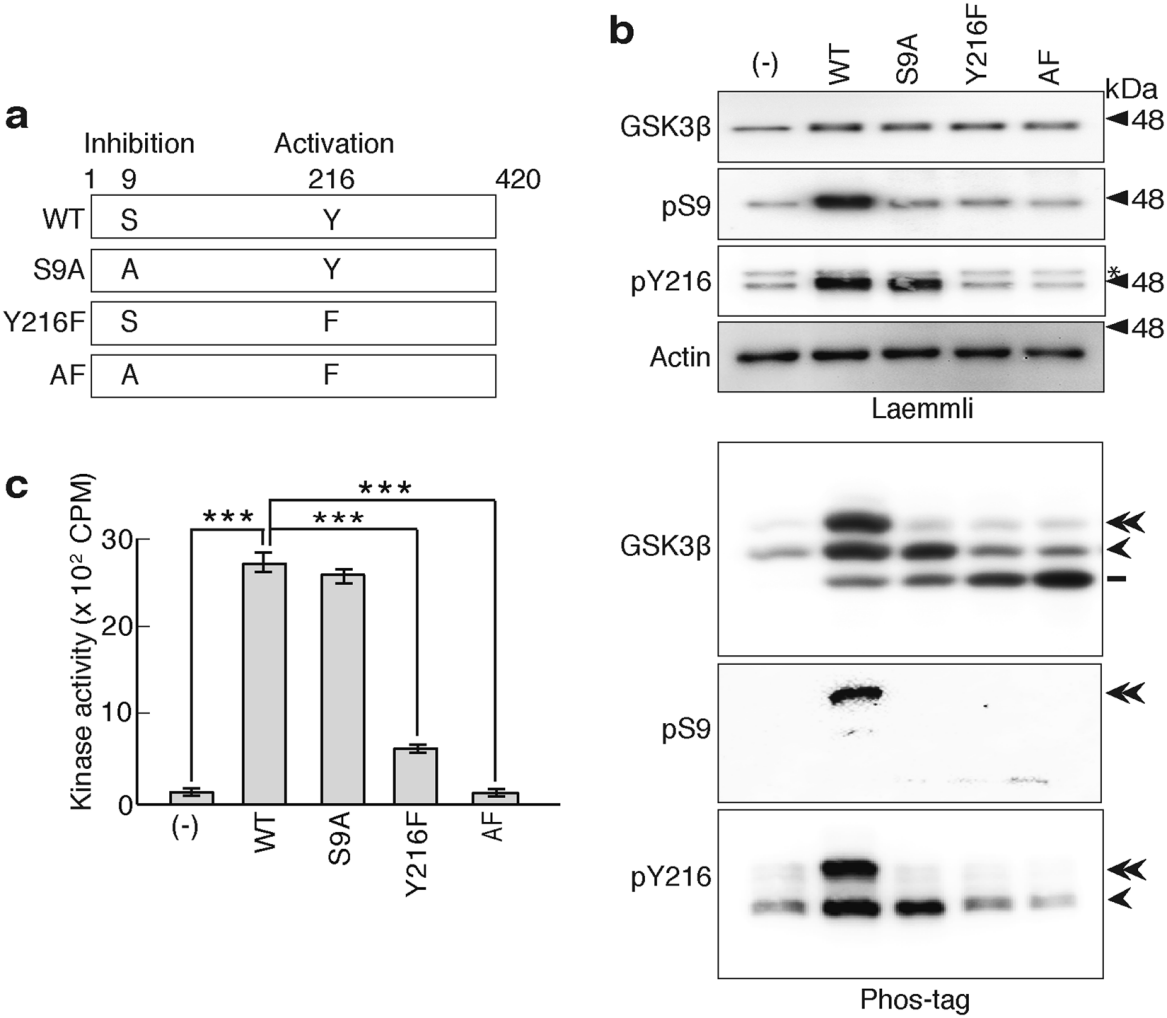
Separation of the three different phosphoisotypes of GSK3β using Phos-tag SDS-PAGE. (**a**) Schematic representation of GSK3β and its mutants at the Ser9 and Tyr216 phosphorylation sites. Ser9, whose phosphorylation inhibits the kinase activity, was mutated to Ala (S9A) and Tyr216, whose phosphorylation is required for the activity, was mutated to Phe (Y216F). GSK3β with a double mutation is indicated by AF. (**b**) Separation of the three GSK3β phosphoisotypes on Phos-tag SDS-PAGE. GSK3β (WT) and its mutants at Ser9 (S9A), Tyr216 (Y216F), or both Ser9 and Tyr216 (AF) were expressed in CHO-K1 cells and subjected to Laemmli’s and Phos-tag SDS-PAGE, followed by immunoblotting with anti-GSK3β, anti-phospho-Ser9 (pS9) and anti-phospho-Tyr216 (pY216) antibodies, as indicated. The left lane shows the control, untransfected cells (-). An asterisk in pY216 blot (third panel) indicates GSK3α. A molecular weight marker of 48 kDa is indicated at the right side of the blots. Actin was used as the loading control in Laemmli’s SDS-PAGE. The amounts of exogenous GSK3β on Phos-tag SDS-PAGE were adjusted prior by immunoblotting with anti-GSK3β after Laemmli’s SDS-PAGE. The phosphorylation states of the three bands of GSK3β on Phos-tag SDS-PAGE are indicated on the right side of the blot; the double arrowhead indicates GSK3β that is phosphorylated at both Ser9 and Tyr216, the arrowhead indicates GSK3β that is phosphorylated at Tyr216, and the bar indicates nonphosphorylated GSK3β. (**c**) Kinase activity of GSK3β and its mutants. The kinase activity was measured with immunoprecipitated GSK3β and its mutants using a GSK3β substrate peptide and [γ-^32^P]ATP (means ± s.e.m. n = 3, the data from one of two independent experiments, ***p < 0.001, one-way ANOVA). Uncropped immunoblots of GSK3β, pS9 and pY216 after Laemmli’s and Phos-tag SDS-PAGE are provided in Supplementary Fig. 5.

We expressed GSK3β in CHO-K1 cells and examined its separation on Phos-tag SDS-PAGE to determine the phosphoisotypes of GSK3β. Although GSK3β appeared as a single band at 47 kDa on Laemmli’s SDS-PAGE gels (Fig. 1b, top panel of Laemmli, WT), it separated into three bands on Phos-tag SDS-PAGE (Fig. 1b, top panel of Phos-tag, WT). Considering that the Phos-tag SDS-PAGE is a phospho-affinity electrophoresis, these three bands should be different phosphorylation states (phosphoisotypes) of GSK3β. We generated Ala and Phe mutants at the two major phosphorylation sites in GSK3β; Ser9 and Tyr216, to determine the phosphorylation states at these sites (Fig. 1a). These mutants were expressed in CHO-K1 cells, and the cell extracts were subjected to Laemmli’s and Phos-tag SDS-PAGE, followed by immunoblotting with anti-GSK3β, anti-phospho-Ser9, and anti-phosphoTyr216 antibodies (Fig. 1b). The S9A mutant displayed two lower bands in the GSK3β blot of Phos-tag. The disappearance of the upper band indicated that the upper band contains phosphorylated Ser9. The Y216F mutation increased the lower band in the GSK3β blot of Phos-tag by diminishing the upper and the middle bands, suggesting that the upper and middle bands contain phosphorylated Tyr216. The double mutation of S9A and Y216F also resulted in the increase of the lower band, indicating the lower band represented nonphosphorylated GSK3β. The faint bands detected at the same positions as exogenous GSK3β were endogenous GSK3β (double and single arrowheads in top panels of Phos-tag), as described below. The specificity of the anti-GSK3β antibody is shown in Supplementary Fig. 1. The anti-GSK3β antibody used here did not react with GSK3α, which appeared above GSK3β (Supplementary Figure 1a).

We immunoblotted the three GSK3β bands on Phos-tag SDS-PAGE with anti-phospho-Ser9 (pS9) or anti-phospho-Tyr216 (pY216) antibodies to confirm the results described above. The anti-phospho-Ser9 antibody only reacted with the upper band and the anti-phospho-Tyr216 antibody recognized both the upper and middle bands (Fig. 1b, lower panels of Phos-tag). Even if the blot of anti-pS9 was exposed for longer time to detect, if any, minor components until nonspecific bands were visible, no other pS9 bands were detected (Supplemental Fig. 2), indicating the upper band alone contains GSK3β with phosphorylation at Ser9. These results convincingly showed that the three different phosphorylation states of GSK3β are separated on Phos-tag SDS-PAGE; the upper band of GSK3β was doubly phosphorylated at Ser9 and Tyr216, the middle band was phosphorylated at Tyr216 and the lower band was not phosphorylated.

It is well known that phosphorylation at Ser9 suppresses the kinase activity and that at Tyr216 is required for the activity^2–4, 12, 13^, indicating that the S9A mutant is fully active and the Y216F mutant should be inactive. We measured the kinase activity of the GSK3β mutants using [γ-^32^P]ATP and a peptide substrate to confirm this hypothesis. Here, we used the peptide substrate with a prime phosphorylation^20^. GSK3β S9A had a similar activity to wild type GSK3β, whereas GSK3β Y216F showed greatly reduced activity and the double mutation expressed the least activity (Fig. 1c). These results indicate the possibility that the measurement of the phospho-Tyr216 isotype by Phos-tag SDS-PAGE provides a simple and appropriate method to estimate the GSK3β activity in cells and tissues.

### Phosphoisotypes of GSK3β in various cell lines

**Figure 2:**
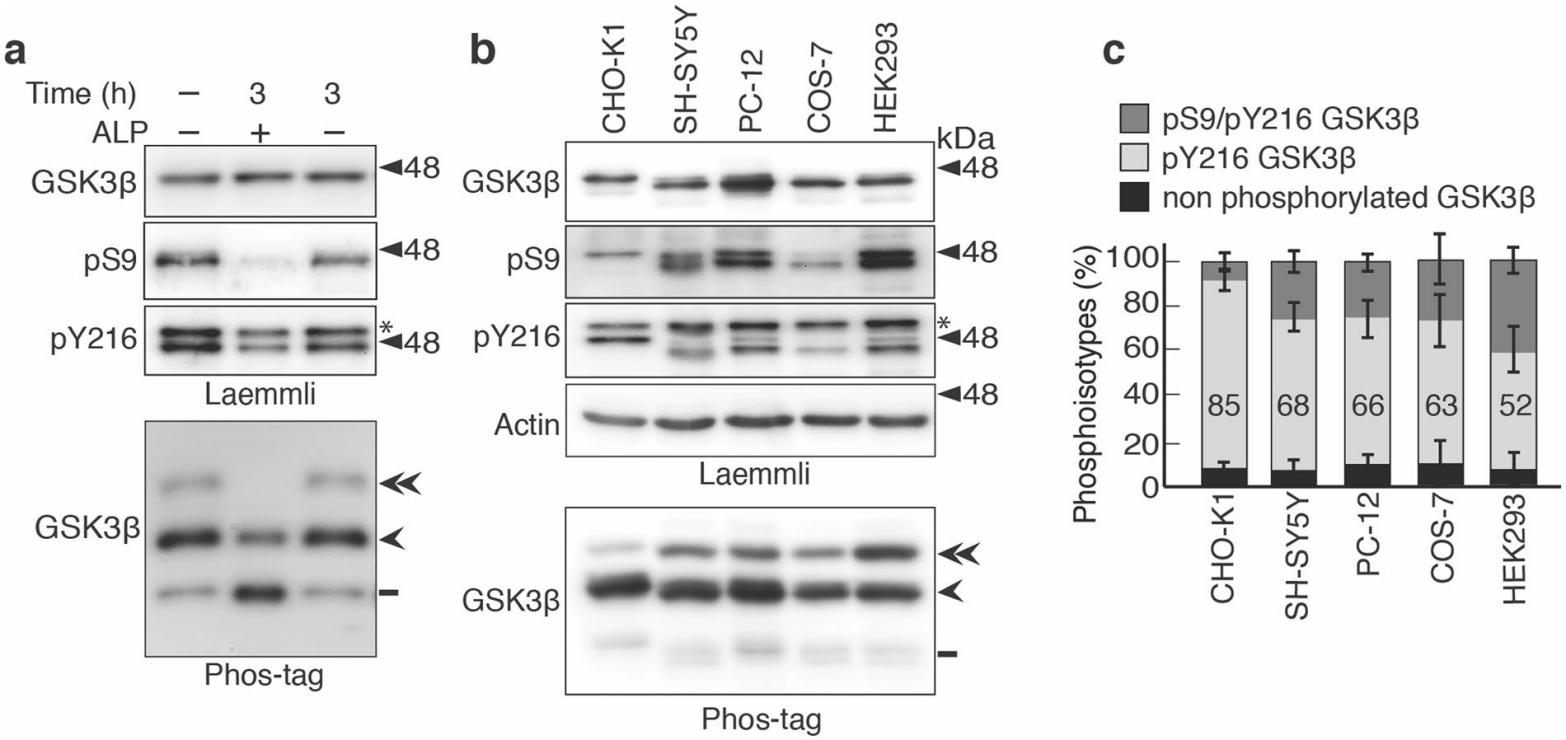
Phosphoisotypes of endogenous GSK3β in various cell lines. (**a**) Dephosphorylation of endogenous GSK3β with alkaline phosphatase (ALP). The CHO-K1 cell extract was incubated with 2.5 U/ml ALP for 3 h and was subjected to Laemmli’s (upper) and Phos-tag (lower) SDS-PAGE, followed by immunoblotting with anti-GSK3β, anti-pS9 and anti-pY216 antibodies. In Phos-tag blot the double-arrowhead indicates Ser9/Tyr216 double phosphorylated GSK3β, the arrowhead indicates Tyr216-phosphorylated GSK3β, and the bar indicates nonphosphorylated GSK3β. (**b**) Immunoblots showing the endogenous GSK3β in cultured cell lines. The cell extracts of CHO-K1, SH-SY5Y, PC-12, COS-7 and HEK293 cells were subjected to Laemmli’s SDS-PAGE, followed by immunoblotting with anti-GSK3β, anti-phospho-Ser9 (pS9) and anti-phospho-Tyr216 (pY216) antibodies. Actin was used as the loading control. An asterisk in the pY216 blot indicates GSK3α. Phos-tag immunoblot of endogenous GSK3β with anti- GSK3β is shown in the bottom panel. The labeling of three bands in Phos-tag is as in (**a**). (**c**) Quantification of the GSK3β phosphoisotypes: the upper dark gray section represents phospho-Ser9/phospho-Tyr216, the middle light gray section represents phospho-Tyr216, and the lower black section represents nonphosphorylated GSK3β. The percentages of phospho-Tyr216 are indicated (means ± s.e.m. n = 3). Uncropped immunoblots after Laemmli’s and Phos-tag SDS-PAGE are provided in Supplementary Fig. 6.

We noticed that endogenous GSK3β was also separated into three phosphoisotypes in the total lysate of untransfected CHO-K1 cells (Fig. 1b, left lane). We then analyzed the phosphorylation states of endogenous GSK3β in various cell lines (Fig. 2). At first, we confirmed whether these three bands indeed represent GSK3β with different phosphorylation levels by dephosphorylation of endogenous GSK3β in the CHO-K1 cell extract with alkaline phosphatase. The incubation with alkaline phosphatase abolished the reaction with anti-pS9 and reduced the reaction with anti-pY216 (Fig. 2a, second and third panels), consistent with the results of Phos-tag, in which the upper band disappeared and the middle bands was reduced (Fig. 2a, lower panel). Further, it is shown that the upper and middle bands of Phos-tag SDS-PAGE are recognized specifically with anti-phospho-Ser9 and anti-phospho-Tyr216 antibodies by the side-by-side immunoblot (Supplementary Fig. 2). Taken together, these results indicate that the upper band corresponds to pS9/pY216-phosphorylated GSK3β, the middle is pY216 GSK3β and the lower band is composed of nonphosphorylated GSK3β.

Next we examined the phosphoisotypes of GSK3β in five different cell lines: CHO-K1, SH-SY5Y, PC-12, COS-7 and HEK293. These cells have been frequently used for various cellular signaling studies. Similar levels of GSK3β were expressed in these cells with higher expression in PC-12 cells (Fig. 2b, top panel of Laemmli). Two closely separated bands were detected with anti- GSK3β and more distinctly with anti-phospho-Ser9 in several cultured cell lines (Fig. 2b, top and second panels of Laemmli). The upper band may be GSK3β2, the alternative spliced variant of GSK3β with a 13 amino acid-insertion^21^. Endogenous GSK3β was similarly separated into three bands on Phos-tag SDS-PAGE in all five cell lines (Fig. 2b). However, the ratio of each phosphoisotype was different when normalized to total GSK3β. The percentages of the phosphoisotypes were measured by densitometric scanning (Fig. 2c). Because these three bands were detected with a single phospho-independent antibody to GSK3β, the measured ratio would directly represent the percentage of each GSK3β phosphoisotype. The Tyr216-phosphorylated, active form was the most abundant, and the highest percentage was 85% in CHO-K1 cells and the lowest was 52% in HEK293 cells. Ser9-phosphorylated, inactive GSK3β also varied from the smallest percentage of 8% in CHO-K1 cells to the maximum of 40% in HEK293 cells. The percentage of nonphosphorylated, inactive GSK3β ranged from 5 to 10% among the cells. These results indicate that half or more of GSK3β molecules are present in the Tyr216-phosphorylated form in cultured cells.

### Effects of GSK3β inhibitors on the phosphorylation states of GSK3β

**Figure 3:**
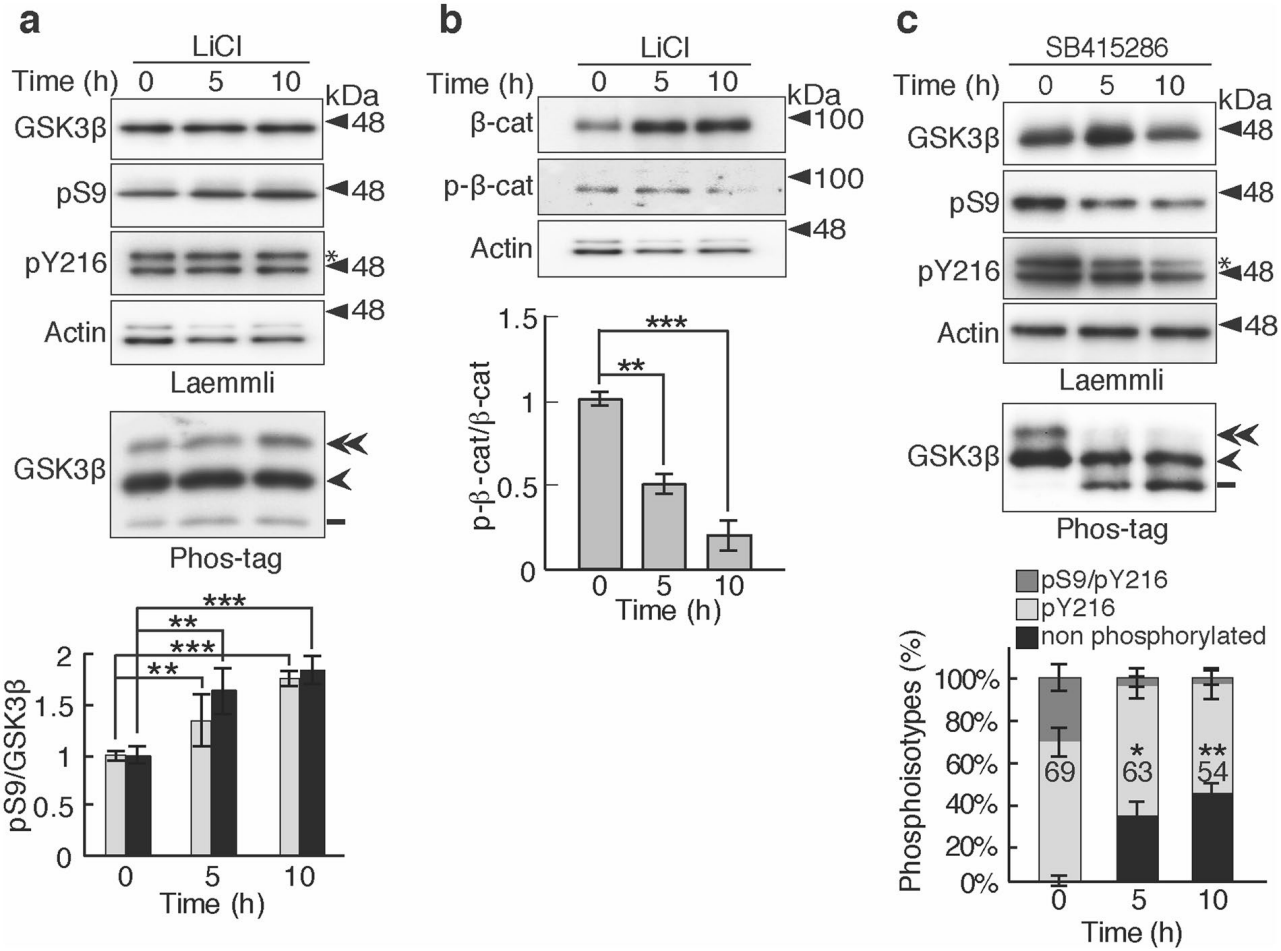
Effects of LiCl and SB415286 on the phosphorylation states of GSK3β. (**a**) Effects of LiCl on the phosphorylation states of GSK3β. CHO-K1 cells were treated with 10 mM LiCl for 5 or 10 h and the resulting cell extracts were subjected to Laemmli’s and Phos-tag SDS-PAGE, followed by immunoblotting with the indicated antibodies. An asterisk in the pY216 blot indicates GSK3α. In Phos-tag (bottom), the double-arrowhead represents Ser9/Tyr216-phosphorylated GSK3β, the arrowhead represents Tyr216-phosphorylated GSK3β, and the bar represents nonphosphorylated GSK3β. Quantification of phospho-Ser9 was shown in the lower graph; gray represents the data from Laemmli and black represents the data from Phos-tag SDS-PAGE (means ± s.e.m. n = 6, **p < 0.01, ***p < 0.001, one-way ANOVA). (**b**) Phosphorylation of β-Catenin in CHO-K1 cells treated with LiCl as in (**a**). The CHO-K1 cell extracts were immunoblotted with anti-β-Catenin or anti- phospho-β-Catenin. Actin was the loading control, the same as shown in (**a**). The ratio of phospho-β-Catenin to total β-Catenin was quantified and expressed against that of the control at 0 h (means ± s.e.m. n = 6, ***p < 0.001, one-way ANOVA). (**c**) Effects of SB415286 on the phosphorylation states of GSK3β. CHO-K1 cell extracts treated with 20 μM SB415286 for 5 or 10 h were subjected to Laemmli’s (upper four panels) and Phos-tag (bottom) SDS-PAGE, followed by immunoblotting with the indicated antibodies. An asterisk in the pY216 blot indicates GSK3α. In Phos-tag, the phosphoisotypes are indicated as in (**a**) and are quantified: the upper dark gray section represents phospho-Ser9/phospho-Tyr216, the middle light gray section represents phospho-Tyr216, and the lower black section represents nonphosphorylated GSK3β. The percentages of phospho-Tyr216 are indicated (means ± s.e.m. n = 6, *p < 0.05, **p < 0.01, one-way ANOVA). Uncropped immunoblots after Laemmli’s and Phos-tag SDS-PAGE are provided in Supplementary Fig. 7.

LiCl and SB415286 are well-known inhibitors of GSK3β with distinct inhibitory mechanisms. Lithium directly inhibits GSK3β by competing with Mg^2+^ ions at the cationic binding site of GSK3β or indirectly through Ser9 phosphorylation^22^, whereas SB415286 competes with ATP at the active site^23, 24^. Here, we tested the effect of LiCl and SB415286 on GSK3β activity by examining the phosphorylation states separated on Phos-tag SDS-PAGE. CHO-K1 cells were treated with 10 mM LiCl or 20 μM SB415286 for 5 or 10 h. LiCl did not change the phosphorylation at Tyr216 significantly on both Laemmli’s and Phos-tag SDS-PAGE, whereas phosphorylation at Ser9 was increased slightly. Even if the incubation with LiCl was prolonged to 24 h, the extent of phospho-Ser9 did not increase further (Supplementary Fig. 3). We confirmed if lithium inhibited GSK3β activity with β-Catenin phosphorylation. Phospho-β-Catenin was reduced with the concomitant increase of its protein amount by LiCl treatment (Fig. 3b). In contrast, SB415286 reduced phosphorylation at both Tyr216 and Ser9 with incubation time, resulting in the increase of nonphosphorylated GSK3β. These results suggest that lithium inhibits GSK3β activity directly by competing with Mg^2+^ ions, although the increased Ser9 phosphorylation may contribute a little, and SB415286 inhibits GSK3β activity by blocking Tyr216 autophosphorylation in addition to the phosphorylation of its substrate proteins^23–25^.

### GSK3β activity estimated by the Phos-tag method and kinase assay

**Figure 4:**
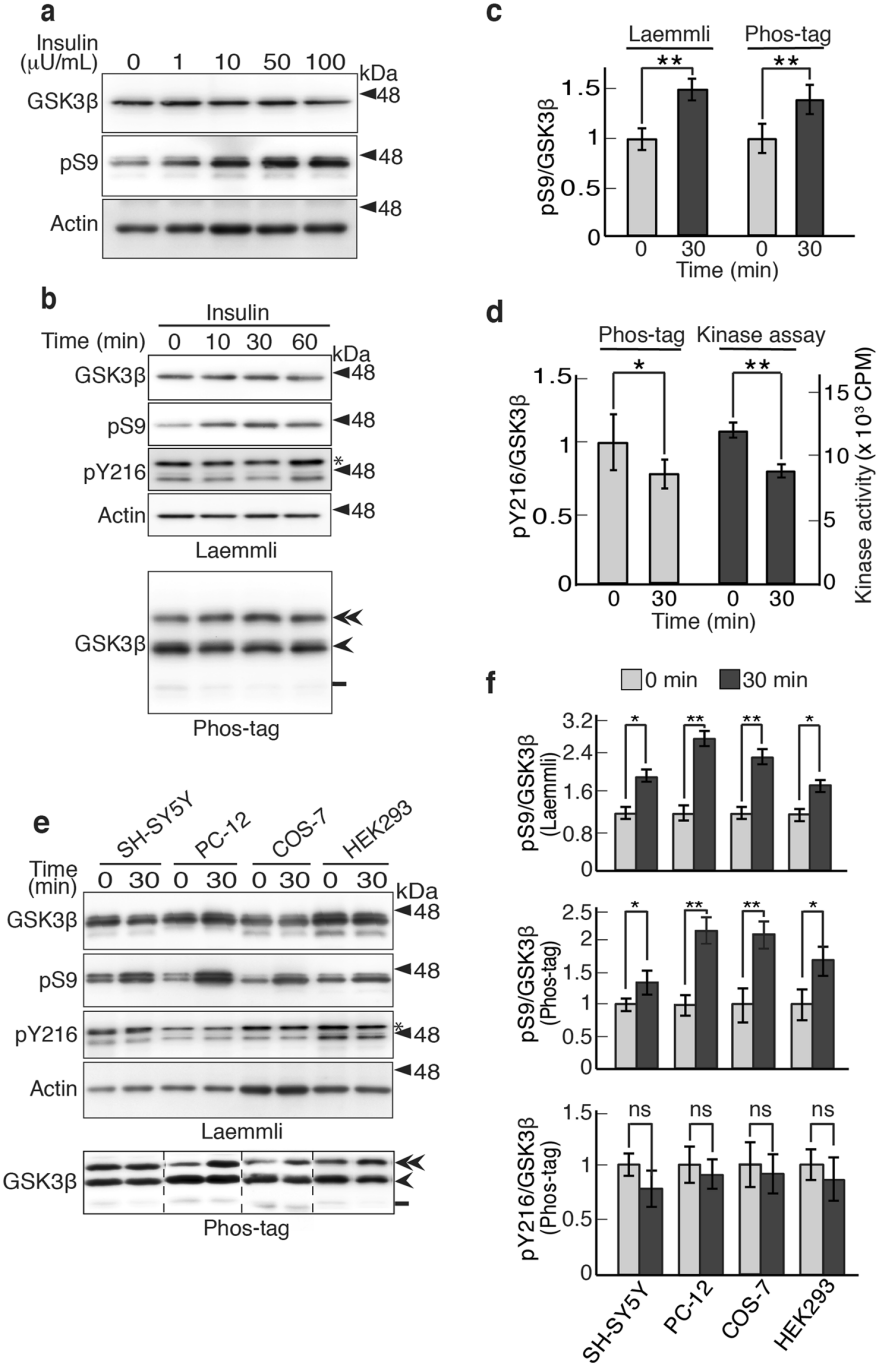
Comparison of GSK3β activity measured using the kinase assay, the anti-phospho-Ser9 antibody and Phos-tag SDS-PAGE. (**a**) Effect of insulin on Ser9 phosphorylation in HEK293 cells. The cells were treated with 0–100 μU/ml insulin for 30 min. The lysates were immunoblotted with anti-GSK3β and phospho-Ser9 (pS9) antibodies. Actin is the loading control. (**b**) Time course of Ser9 phosphorylation of GSK3β in HEK293 cells treated with 50 μU/ml insulin for the indicated times. The upper four panels are immunoblots with the indicated antibodies after Laemmli’s SDS-PAGE and the bottom panel shows a Phos-tag immunoblot with anti-GSK3β. An asterisk in the pY216 blot indicates GSK3α. Double arrowhead, Ser9/Tyr216-phosphorylated GSK3β; arrowhead, Tyr216-phosphorylated GSK3β; bar, nonphosphorylated GSK3β. **(c)** Quantification of the phospho-Ser9 (pS9) levels. The ratio of the pS9 to total GSK3β was measured by the anti-phospho-Ser9 reaction on Laemmli’s SDS-PAGE (left) and the phospho-Ser9 band on Phos-tag SDS-PAGE (right) and expressed as the ratio to the control at 0 min. (**d**) Comparison of the phospho-Tyr216 (pY216) levels with the kinase activity of GSK3β. The ratio of pY216 to total GSK3β as measured in the lysates of HEK293 cells treated with 50 μU/ml insulin for 0 and 30 min using the Phos-tag method. Kinase activity in immunoprecipitated GSK3β was measured using [γ-^32^P]ATP and the peptide substrate (means ± s.e.m. n = 3, *p < 0.05, **p < 0.01, Student’s *t* test). (**e**) Effect of insulin on Ser9 phosphorylation of GSK3β. SH-SY5Y, PC-12, COS-7 or HEK293 cells were treated with insulin for 30 min. The upper four panels show Laemmli’s and the lower panel shows Phos-tag SDS-PAGE. Phos-tag blots of respective cell extracts were carried out separately with the same sample sets used for Laemmli’s blots. Phosphoisotypes are indicated as in (**b**). Actin is the loading control. An asterisk in the pY216 blot indicates GSK3α. (**f**) Quantification of the phospho-Ser9 (pS9) and phospho-Tyr216 (pY216) levels. The ratio of pS9 to total GSK3β was measured by the anti-phospho-Ser9 reaction on Laemmli’s SDS-PAGE (upper) and the double-phosphorylated GSK3β bands (double arrowhead) on Phos-tag SDS-PAGE (middle) and expressed as the ratio to that in respective untreated control cells. The GSK3β bands with single phosphorylation at Tyr216 (arrowhead) were quantified and expressed similarly as the ratio to that in respective untreated control cells (lower) (means ± s.e.m. n = 3, *p < 0.05, **p < 0.01, ns, not significant, Student’s *t* test). Uncropped immunoblots after Laemmli’s and Phos-tag SDS-PAGE are provided in Supplementary Fig. 8.

GSK3β is constitutively active and is suppressed by phosphorylation at Ser9 by several protein kinases, including Akt, a kinase downstream of insulin signaling^2–4^. Inhibition of GSK3β activity has been evaluated by increased Ser9 phosphorylation^26, 27^. We assessed whether the measurement of phospho-Tyr216 by Phos-tag can be used as a simple method to precisely evaluate the GSK3β activity. First, we treated HEK293 cells with 0-100 μU/ml insulin for 30 min (Fig. 4a). The maximum increase in Ser9 phosphorylation was obtained at 50 μU/ml insulin. Then, the cells were treated with 50 μU/ml insulin for the indicated times (Fig. 4b). Inhibitory phosphorylation at Ser9 reached plateau level at 30 min, consistent with previous reports^2–4, 26, 27^. Accordingly, we determined the incubation time 30 min in the following experiments. The changes in the phosphorylation states were observed using Phos-tag SDS PAGE after normalization with total GSK3β (Fig. 4b, lower panel). The GSK3β activity levels were quantified in several different ways: Ser9 phosphorylation, Tyr216 phosphorylation and the kinase assay. Ser9 phosphorylation was increased up to approximately 150%, when it was estimated by blotting with an anti-phospho-Ser9 after Laemmli’s SDS-PAGE (Fig. 4c, left), and a similar increase of 140% was detected in the phospho-Ser9 band on Phos-tag SDS PAGE (Fig. 4c, right). On the other hand, the phospho-Tyr216 band decreased by 25% when evaluated by Phos-tag (Fig. 4d, left). We measured the activity of GSK3β using conventional kinase assay with a peptide substrate with the prime phosphorylation and [γ-^32^P]ATP to verify if phospho-Tyr216 indeed represents the GSK3β activity. The insulin treatment decreased the GSK3β kinase activity by 27% (Fig. 4d, right), which was fairly consistent with the phospho-Tyr216 levels detected by the Phos-tag technique.

Because the amount of phospho-Tyr216 can serve well as a substitute for the kinase activity, we used it to evaluate the insulin-dependent inhibition of GSK3β in several cell lines. The cells were treated with 50 μU/ml insulin for 30 min, and the cell extracts were subjected to Laemmli’s and Phos-tag SDS-PAGE and immunoblotted with anti-phospho-Ser9 and anti-GSK3β antibodies, respectively. The treatment increased the phospho-Ser9 levels in all cell lines when it was measured by either Laemmli’s (Fig. 4e, upper panels) or Phos-tag SDS-PAGE (Fig. 4e, bottom panel). The 0.75–2-fold increase in phospho-Ser9 level quantified using phospho-Ser9 specific antibody in Laemmli’s (Fig. 4f, upper) corresponded to that quantified with phospho-Ser9 band in Phos-tag SDS-PAGE (Fig. 4f, middle). The decrease in phospho-Tyr216 levels was also quantified using phospho-Tyr216 band in Phos-tag SDS-PAGE (Fig. 4f, lower), but the levels in decrease were 5–20% similar to those measured by phospho-Tyr216 blot of Laemmli’s SDS-PGAE, depending on the cell line. These results suggest that only a small fraction of GSK3β is inhibited by insulin treatment, not so much as those expected from the increased phospho-Ser9.

### GSK3β phosphoisotypes in primary cortical neurons

**Figure 5:**
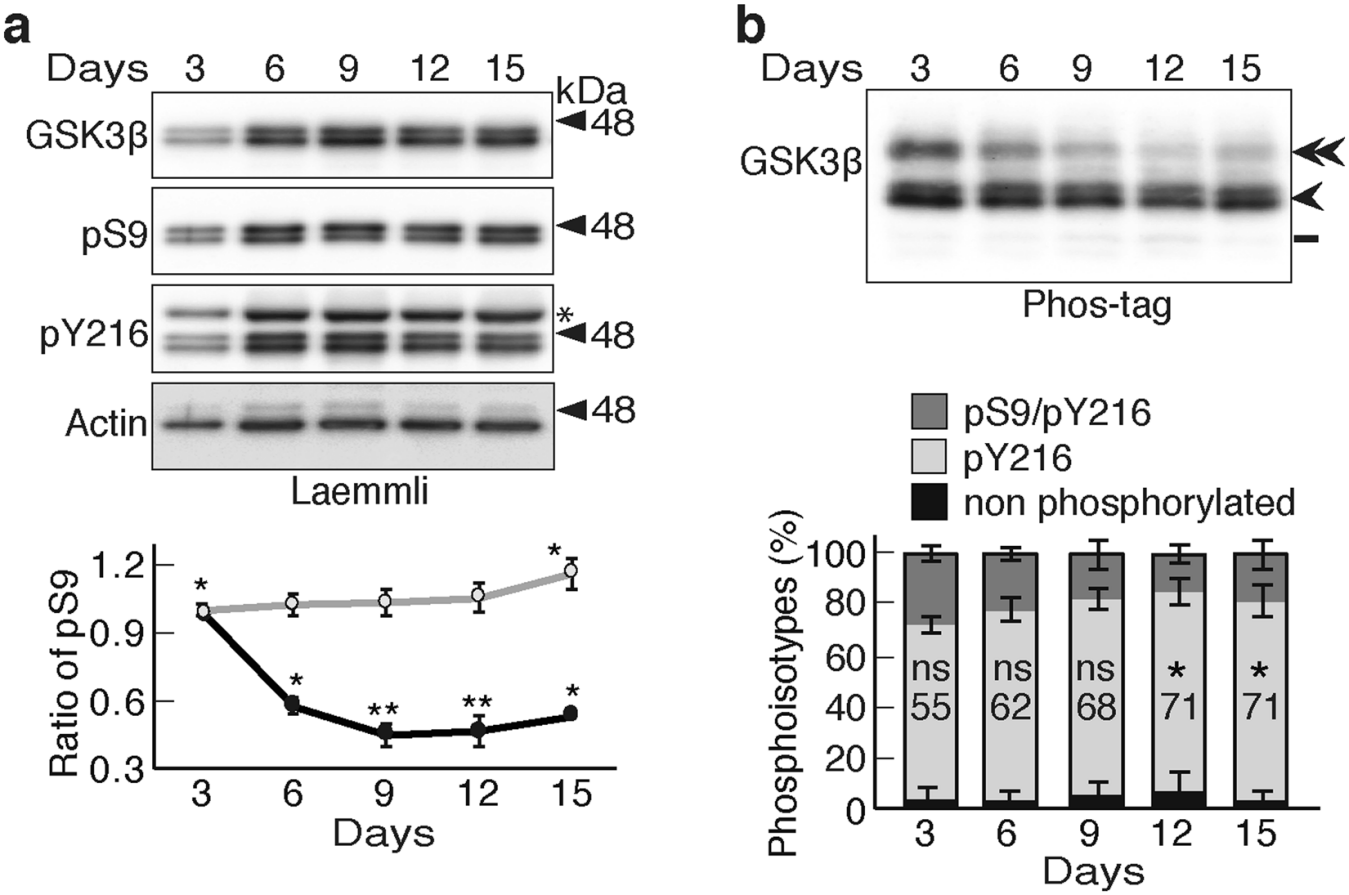
GSK3β phosphoisotypes in primary neurons. (**a**) Immunoblotting of mouse brain cortical neurons cultured for 3, 6, 9, 12 and 15 DIV with indicated antibodies after Laemmli’s SDS-PAGE. An asterisk in the pY216 blot indicates GSK3α. Quantification of the phospho-Ser9 levels normalized to the GSK3β (black) or actin (gray) levels is shown in the lower graph (means ± s.e.m. n = 6, *p < 0.05, **p < 0.01, one-way ANOVA) (**b**) Immunoblotting of the neuron extracts with an anti-GSK3β antibody after Phos-tag SDS-PAGE. The amount of the samples applied was adjusted with total GSK3β. The ratios of three phosphoisotypes of GSK3β are shown: dark gray, phospho-Ser9/phospho-Tyr216; light gray, phospho-Tyr216; black, nonphosphorylated GSK3β. The percentages of phospho-Tyr216 are indicated (means ± s.e.m. n = 6, *p < 0.05, ns, not significant, one-way ANOVA). Uncropped immunoblots of GSK3β, pS9, pY216 and actin after Laemmli’s SDS-PAGE and GSK3β after Phos-tag SDS-PAGE are provided in Supplementary Fig. 9a.

GSK3β phosphorylate tau protein to produce its pathological epitopes, which are used for diagnosis of AD. However, it is not answered yet why GSK3β-induced abnormal phosphorylation occurs in AD brains. To get insights, we examined GSK3β phosphoisotypes in primary neurons. The expression and phosphorylation of GSK3β at Ser9 and Tyr216 are shown along with the days-*in-vitro* (DIV) in culture (Fig. 5a). GSK3β2 was also detected. The expression levels of total GSK3β, GSK3β1 and GSK3β2, reached a plateau at 6 DIV and remained constant for approximately next 8 days (Fig. 5a, top panel). Phospho-Tyr216 also showed almost constant expression from 6 DIV. An upper band in the blot of pY216 (indicated by asterisk) is GSK3α (Fig. 5a). The phospho-Ser9 levels appeared to be constant or increase a little when the bands were normalized to actin (Fig. 5a, lower graph, gray). However, when normalized to total GSK3β levels, the phospho-Ser9 levels were the highest at 3 DIV and then decreased (Fig. 5a, lower graph, black). The phosphoisotypes were examined using the Phos-tag method (Fig. 5b, normalized with GSK3β). Although the middle band of phospho-Tyr216 was detected as a doublet, the upper phospho-Ser9/phospho-Tyr216 band appeared as a single band, probably due to the insufficient separation of double phosphorylated bands. Therefore, we quantified the GSK3β1 and GSK3β2 bands together. It is clear that the phospho-Ser9 levels were the highest at 3 DIV and gradually decreased with culturing until 12 DIV. In contrast, the GSK3β activity represented by phospho-Tyr216 increased gradually with the DIV (Fig. 5b, lower graph).

### Effect of IGF-1 on GSK3β activity in cultured primary neurons estimated using the Phos-tag method

**Figure 6:**
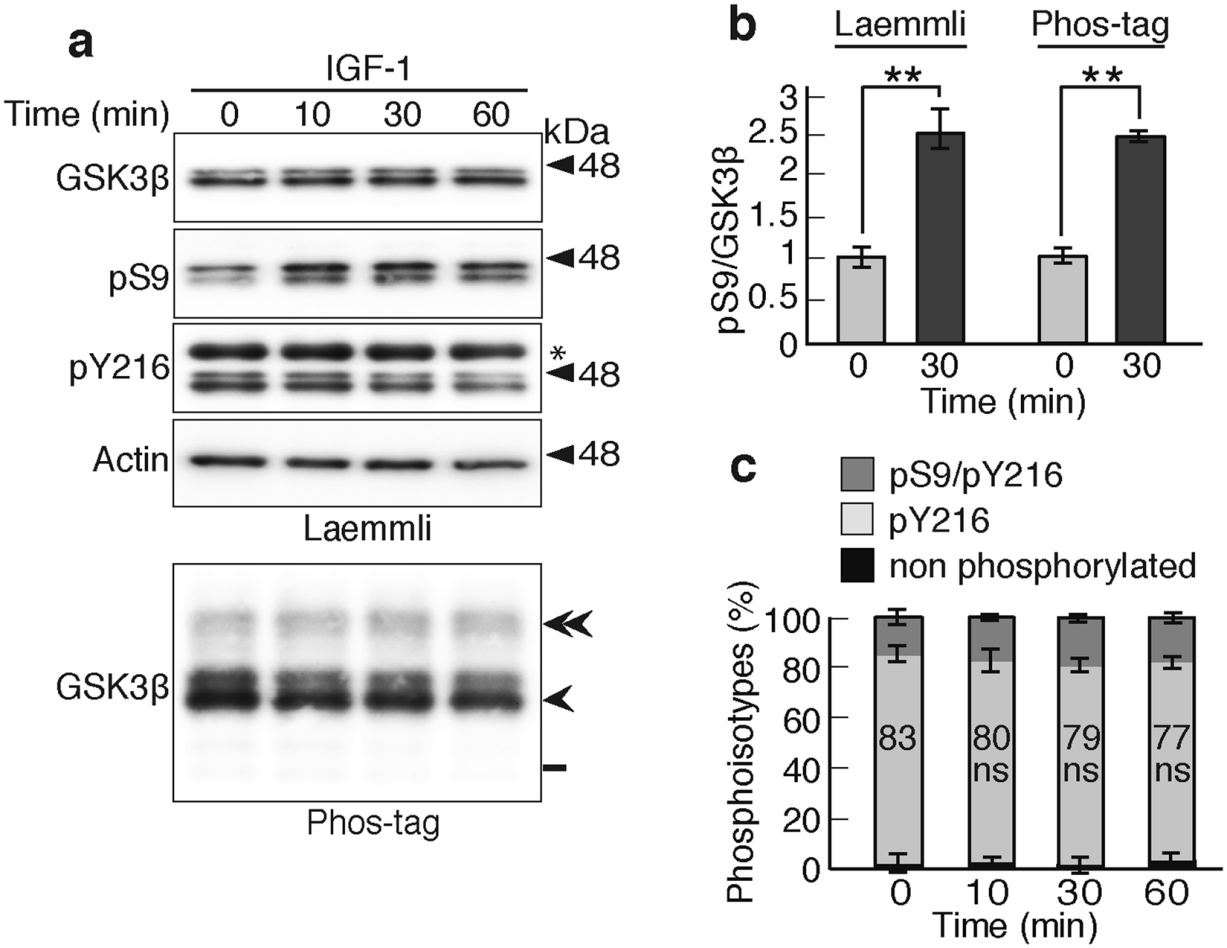
Effect of IGF-1 on GSK3β activity in cultured primary neurons. (**a**) Effect of IGF-1 on Ser9 phosphorylation of GSK3β in mouse cortical neurons. Neurons in culture at 5 DIV were treated with 100 ng/ml IGF-1 for the indicated times, and the extracts were subjected to Laemmli’s SDS-PAGE, followed by immunoblotting with an anti-GSK3β, anti-phospho-Ser9 and anti-phospho-Tyr216 or Phos-tag SDS-PAGE, followed by immunoblotting with the anti-GSK3β. Actin is the loading control. An asterisk in the pY216 indicates GSK3α. Double arrowhead, Ser9/Tyr216-phosphorylated GSK3β; arrowhead, Tyr216-phosphorylated GSK3β; bar, nonphosphorylated GSK3β. (**b**) Levels of pSer9 in neurons that were treated with IGF-1 for 30 min. The ratio of pS9/GSK3β was measured and expressed to that of the control at 0 min. The measurements by Laemmli’s and Phos-tag SDS-PAGE are shown in left and right, respectively. (**c**) The quantification of three phosphoisotypes of GSK3β in a Phos-tag blot: dark gray, phospho-Ser9/phospho-Tyr216; light gray, phospho-Tyr216; black, nonphosphorylated GSK3β. The percentages of phospho-Tyr216 are indicated (means ± s.e.m. n = 6, ns, not significant). Uncropped immunoblots of GSK3β, pS9, pY216 and actin after Laemmli’s SDS-PAGE and GSK3β after Phos-tag SDS-PAGE are provided in Supplementary Fig. 9b.

We tested the effect of insulin-like growth factor (IGF) and insulin on GSK3β activity in primary neurons at 5 DIV^28–30^, when the phosphorylation pattern of GSK3β became nearly constant. Since larger response was obtained with IGF-1 than insulin, the data of IGF-1 were shown. The IGF-1 at 100 ng/ml increased the levels of phospho-Akt, indicating the activation of IGF-1 signaling (Supplementary Fig. 4). Inhibitory phosphorylation at Ser9 was increased at 10 min and reached a maximum at 30 min and then decreased slightly at 60 min on IGF-1 treatment, whereas the phosphorylation at Tyr216 was slightly decreased at 30 and 60 min with total GSK3β remaining constant. Interestingly, Ser9 phosphorylation of GSK3β2 was stronger than GSK3β1, although its total amount was much less than GSK3β1. GSK3β2 appeared to respond to IGF-1 more than GSK3β1. Immunoblots of GSK3β after Phos-tag SDS-PAGE (normalized to total GSK3β) are shown in lower panels of Fig. 6a. Quantification revealed an approximate 1.5-fold increase in the ratio of phospho-Ser9 to total GSK3β after IGF-1 treatment on Phos-tag SDS-PAGE (Fig. 6b, right), which was roughly similar to the 1.7-fold increase when estimated with an anti-phospho-Ser9 antibody on Laemmli’s SDS-PAGE (Fig. 6b, left). Nevertheless, the phospho-Tyr216 band accounted for the majority (more than 75%) of GSK3β (Fig. 6c, light gray), greater than those in most of cultured cell lines. The active GSK3β with phospho-Tyr216 maintained almost a constant amount even after the IGF-1 treatment.

### GSK3β phosphoisotypes in mouse brain

**Figure 7:**
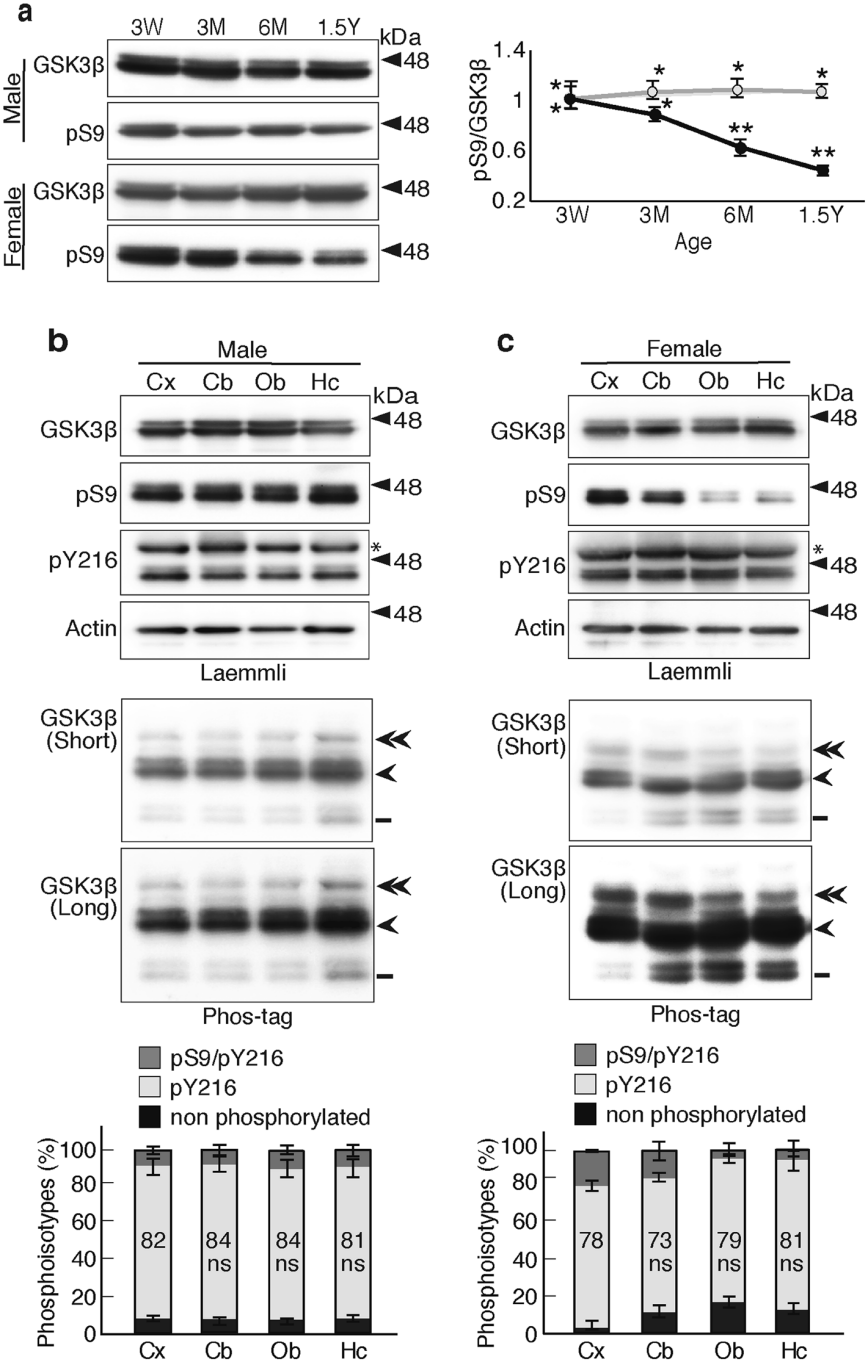
GSK3β phosphoisotypes in mouse brains. (**a**) Expression and Ser9 phosphorylation of GSK3β in mouse brain with aging. Extracts of hippocampus of male and female mouse at ages 3 weeks (3 W), 3 and 6 months (3 M and 6 M), and 1.5 years (1.5Y), were immunoblotted with anti-GSK3β or anti-phospho-Ser9 after Laemmli’s SDS-PAGE. The ratio of phospho-Ser9 to total GSK3β was quantified and expressed to the ratio at 3 weeks. Gray represents male and black represents female (means ± s.e.m. n = 6, *p < 0.05, **p < 0.01, one-way ANOVA). (**b** and **c**) Analysis of GSK3β phosphorylation in different regions of brains prepared from 1.5-year-old mice; cerebral cortex (Cx), cerebellum (Cb), olfactory bulb (Ob) and hippocampus (Hc). (**b**) shows the male mice and (**c**) shows the female mice. The top four panels show Laemmli’s SDS-PAGE and the lower two panels (upper, shorter exposure; lower, longer exposure) show Phos-tag SDS-PAGE that had been immunoblotted with the indicated antibodies. The percentages of three phosphoisotypes of GSK3β are shown below the blots. Dark gray represents phospho-Ser9 and phospho-Tyr216 GSK3β, light gray represents phospho-Tyr216, and black represents nonphosphorylated GSK3β. The percentages of phospho-Tyr216 are indicated (means ± s.e.m. n = 6, ns, not significant). Uncropped immunoblots of GSK3β, pS9, pY216 and actin after Laemmli’s SDS-PAGE and GSK3β after Phos-tag SDS-PAGE are provided in Supplementary Fig. 10.

The degree of phospho-Ser9 was decreased with maturation of cultured neurons (Fig. 5). Then we examined expression of GSK3β and its Ser9 phosphorylation with aging of mouse. The blots of Fig. 7a show the total and phospho-Ser9 GSK3β levels in the hippocampus of male (upper) and female mice (lower) at different ages. The ratio of phospho-Ser9 to total GSK3β was quantified in right. Although phospho-Ser9 levels did not change in the male hippocampus, the levels in the female hippocampus decreased by approximately one-half in 1.5 years compared to the levels at 3 weeks. Thus, the phospho-Ser9 levels in wild type female mice changed with aging. There is a report that describes the age-dependent change in the regulation of GSK3β in p25 Cdk5 activator transgenic mouse brain^31^.

We then analyzed the phosphorylation of GSK3β in brains of aged male and female mouse at age 1.5 years, using four different regions; cerebral cortex, cerebellum, olfactory bulb and hippocampus (Fig. 7b and c). After confirming that the amounts of GSK3β were similar (Fig. 7b and c, top panel), we examined the phosphorylation states with anti-phospho-specific antibodies (Fig. 7b and c, second and third panels) and Phos-tag SDS-PAGE (Fig. 7b and c, fifth panel). The anti-phospho-Tyr216 antibody detected two major bands, which would correspond to GSK3α and GSK3β2/1, respectively. In male mouse brain, approximately 80% of GSK3β was phosphorylated at Tyr216 (Fig. 7b, lower panel). The blots indicated that there was only a minor variation at the expression levels of GSK3β and its phosphorylation among brain regions. Interestingly, in female mice at the same age, the anti-phospho-Ser9 antibody displayed a weaker reaction to GSK3β in the olfactory bulb and hippocampus than the cerebral cortex and cerebellum (Fig. 7c, second panel). The reduced phospho-Ser9 levels in the olfactory bulb and hippocampus were confirmed by Phos-tag method (Fig. 7c, fifth panel). Conversely, the levels of nonphosphorylated GSK3β were increased by 17% in the olfactory bulb and 13% in the hippocampus, when compared with that in the cerebral cortex (Fig. 7c, Phos-tag). Therefore, the amounts of phospho-Tyr216 GSK3β were fairly constant in these regions, ranging from 73% in the cerebellum to 81% in the hippocampus in female mouse brain (Fig. 7c, bottom panel). This is the first evidence indicating that phospho-Ser9 levels are differently regulated depending on sex, age and brains regions of mouse.

### GSK3β phosphoisotypes in diabetic mouse brain

**Figure 8:**
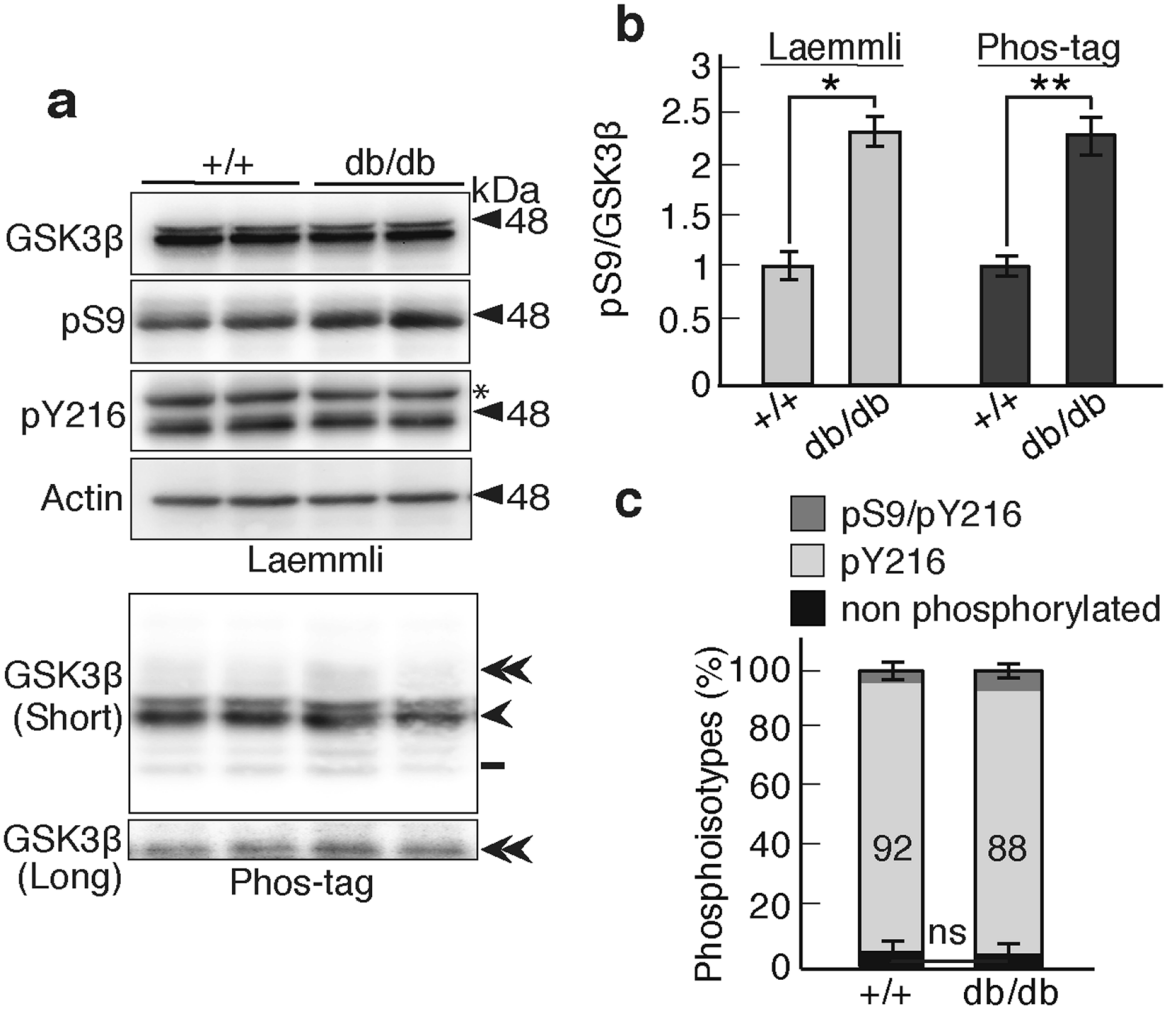
GSK3β phosphoisotypes in the brains of db/db mice. (**a**) GSK3β, phospho-Ser9 (pS9), phospho-Tyr216 (pY216) and actin in the cerebral cortex of wild type mice (+/+, left two lanes) or db/db (right two lanes) were examined using the Laemmli’s SDS-PAGE and phosphorylation of GSK3β by Phos-tag method. (**b**) The ratio of phospho-Ser9 to total GSK3β in wild type (+/+) and db/db mice. The left two bars show the data obtained from the anti-phospho-Ser9 immunoblot after Laemmli’s SDS-PAGE and the right two bars show the data obtained with Phos-tag SDS-PAGE. The ratio is expressed relative to that in the wild type (+/+) mouse brain (means ± s.e.m. n = 4, *p < 0.05, **p < 0.01, Student’s *t* test). (**c**) The percentages of the GSK3β phosphoisotypes on Phos-tag SDS PAGE were quantified: dark gray, phospho-Ser9/Tyr216; light gray, phospho-Tyr216; and black, nonphosphorylated GSK3β. The percentages of phospho-Tyr216 are indicated (means ± s.e.m. n = 4, ns, not significant). Uncropped immunoblots of GSK3β, pS9, pY216 and actin after Laemmli’s SDS-PAGE and GSK3β after Phos-tag SDS-PAGE are provided in Supplementary Fig. 11.

It is suggested that diabetes is linked to higher risk of AD^32, 33^. Then, we analyzed the GSK3β phosphoisotypes in the brains of db/db mice, a model of Type 2 diabetes^33^, using the Phos-tag method. The db/db mouse displays hyperglycemia and insulin-resistance. There are three reports on GSK3 activity in the db/db mouse brain with different results: increased Ser9 phosphorylation^33^ and no change in phosphorylation^34^. It is recently reported that Ser9 phosphorylation increased at 4 weeks, but not 26 weeks in db/db mice^35^. The phosphoisotypes were examined in the brains of 11-week-old male mice using Laemmli’s (Fig. 8a, upper four panels) or Phos-tag SDS-PAGE (Fig. 8a, bottom panels). When the samples were normalized to actin, the total GSK3β levels in the non-diabetic WT and db/db mice were not different. When normalized to the total GSK3β levels, Ser9 phosphorylation was slightly but significantly increased in the db/db mice compared to the WT animals (Fig. 8b, left). A similar increase was observed using the Phos-tag method (Fig. 8b, right). The levels of Tyr216-phosphorylated GSK3β were also measured as approximately 92% in wild type C57BL/6 mice (Fig. 8c). In the db/db mice, the ratio of phospho-Ser9 to total GSK3β were increased 1.25-fold (Fig. 8b, right panel), but the Tyr216-phosphorylated GSK3β levels were decreased by only 4% when estimated with the phospho-Tyr216 band on Phos-tag. Thus, even if GSK3β is inhibited in the db/db mouse brain, the level of inhibition would be very low.

## Discussion

The kinase activity of GSK3β has mainly been estimated by the degree of inhibitory phosphorylation at Ser9, such that GSK3β should be inhibited because Ser9 phosphorylation is increased. However, it has also been noted that Ser9 phosphorylation is not an accurate indicator of GSK3β activity, and a method for properly measuring GSK3β activity *in vivo* has long been expected. Recently, the antibodies recognizing non-phosphorylated Ser9 were reported^36^. Here, we developed a method for estimating the kinase activity of GSK3β using Phos-tag SDS-PAGE. This method is as simple as the detection of Ser9 phosphorylation by immunoblotting and can also be applied to GSK3β *in vivo*. Using this method, we showed a number of novel insights into GSK3β activity, including that GSK3β activity is differentially regulated in brains, depending on the regions, ages, sex, and disease conditions.

GSK3β was separated into three phosphoisotypes on Phos-tag SDS-PAGE, Ser9 and Tyr216 double phosphorylated, Tyr216 single-phosphorylated and nonphosphorylated GSK3β. Most GSK3β was phosphorylated at Tyr216, consistent with the notion that Tyr216 phosphorylation occurs cotranslationally by autophosphorylation^10^. A minor population of nonphosphorylated GSK3β was first detected using this method, which is capable of separating nonphospho-proteins from their phosphorylated counterparts. The nonphosphorylated form must have either escaped from autophosphorylation during translation or been produced by dephosphorylation after it was initially phosphorylated. We think that both can occur. The SB415286 treatment increased the amount of nonphosphorylated GSK3β in cells. This result suggests that the inhibitor works to inhibit the auto- and Tyr-phosphorylation of naïve GSK3β and that full length GSK3β is translated even if the autophosphorylation process is skipped. On the other hand, a distinct amount of nonphosphorylated GSK3β was detected in the hippocampus, cerebellum and a maximum of ~20% at olfactory bulb of aged female mouse brains, compared to merely 5% in cortex of the same female mouse or brains of male mouse where nonphosphorylated GSK3β was marginally detected. It seems more likely that this variability is caused by dephosphorylation of autophosphorylated GSK3β rather than a translational event. Although this hypothesis must be investigated in the future, the results suggest that GSK3β activity is regulated by phosphorylation and dephosphorylation of Tyr216 in brain.

There were only three GSK3β phosphoisotypes in cultured cells. We did not detect single Ser9-phosphorylated band of GSK3β. Ser9 phosphorylation was neither observed with Y216F mutant. Furthermore, Ser9 phosphorylation was decreased as Tyr216 phosphorylation decreased in cells that were treated with SB415286. It might be difficult to detect phospho-Ser9 GSK3β, even if the reaction occurred, because of a minor population of nonphosphorylated GSK3β in cells, but these results likely suggest that Tyr216 phosphorylation may be required for Ser9 phosphorylation. Phosphorylation at Tyr216 may change the conformation of the N-terminal tail region of GSK3β to confer the regulation by Ser9 phosphorylation of GSK3β. Phosphorylation on Ser43 and Ser389/390 has also been reported for GSK3β^37, 38^. We did not examine phosphorylation at these sites by mutating the sites because we did not identify other phosphoisotypes in the cells that might correspond to these phosphoisotypes. However, in neurons or brain extracts, there were minor, unidentified anti-GSK3β-reactive bands, some of which might be GSK3β with phosphorylation at the above sites, because it was reported that Ser389/390 phosphorylation primarily occurs in brains^38^.

It was interesting to observe the different inhibitory actions of lithium and SB415286 using Phos-tag method. Lithium has been shown to directly inhibit GSK3β activity by competing with Mg^2+^ or indirectly by enhancing Akt activity and/or inhibiting phospho-Ser9 phosphatase activity^22, 39, 40^. Phospho-Tyr216 levels did not change in lithium-treated cells, whereas phospho-Ser9 was increased a little, supporting the idea that lithium inhibits GSK3β activity mainly by competition with Mg^2+^ binding but not through Ser9 phosphorylation in cells. However, this inhibition does not affect the autophosphorylation of Tyr216 as was observed with SB415286. SB415286 has been shown to inhibit GSK3β by competing with ATP^23, 24^. SB415286 increased the levels of the inactive, nonphosphorylated form of GSK3β. This result can be explained if SB415286 inhibits autophosphorylation activity. SB415286 would inhibit GSK3β by inhibiting its activity toward other substrate proteins but also prevents the activation of GSK3β itself. Thus, the different inhibitory mechanisms between lithium and SB415286 are clearly validated by the use of Phos-tag method.

It is accepted that cellular GSK3β is active upon phosphorylation at Tyr216 in the activation loop^2–4, 41, 42^. We confirmed this in the present study using an *in vitro* kinase assay with immunoprecipitated GSK3β or its phosphorylation site mutants. Mutation of Tyr216 to Phe nearly abolished the kinase activity. Exogenous or endogenous GSK3β was most abundantly expressed as the Tyr216-single phosphoisotype in cells, confirming that the majority of GSK3β in cells is active in terms of both the phosphorylation state and the kinase activity^41, 42^. Ser9 phosphorylation inactivates GSK3β and the degree of Ser9 phosphorylation has been employed as a marker for cellular inhibition of GSK3β^2–4, 12, 13, 41, 42^. Using the Phos-tag method, we measured the percentage of phospho-Ser9 GSK3β in cells and found that Ser9 phosphorylation was relatively low with some variations depending on types or conditions of the cells. The lowest was ~8% in CHO-K1 cells and the highest was ~40% in HEK293 cells. Ser9 is phosphorylated by several protein kinases in response to stimulation^12, 13^. The differences in Ser9 phosphorylation may represent the robustness of these signaling cascades in the respective cells. In cultured primary neurons, phospho-Ser9 was more than 30% at 3 DIV and reduced to 15% at 12 DIV. Higher Ser9 phosphorylation levels, that mean lower GSK3β activity, in early developmental stages could be a remnant of the lower actively proliferating progenitor cells^43^. In general, the levels of phospho-Ser9 were very low in matured neurons, less than 10% of total GSK3β in the adult male mouse brains. In contrast, higher levels of Ser9 phosphorylation were detected in the cerebral cortex (approximately 25%) and cerebellum of aged female mouse. These results could also be observed clearly and firstly by the use of the Phos-tag method. The differences of GSK3β phosphoisotypes between sexes or among brain regions may be related to age-dependent changes in hormone levels, such as estradiol or insulin-like growth factor^43, 44^.

Akt, a kinase downstream of insulin signaling, is most widely investigated as a GSK3β-inhibitory kinase^2–4, 12, 13, 41, 42^. We treated cells with insulin to determine the extent of the increased Ser9 phosphorylation of GSK3β. Consistent with previous many reports, Ser9 phosphorylation increased approximately 0.5-fold upon insulin treatment when it was examined by immunoblotting with the anti-phospho-Ser9 antibody. A similar extent of increased phosphorylation was obtained, even when the upper Ser9-phospho-band in Phos-tag was measured, indicating that the Phos-tag method measures the relative changes of Ser9 phosphorylation properly. However, the increase was rather small when the proportion of phospho-Ser9 GSK3β was evaluated against the total GSK3β molecule. For example, in PC-12 cells, the population of GSK3β with Ser9 phosphorylation was increased almost 2-fold by insulin treatment (Fig. 4e and upper panel of Fig. 4f). However, it represents only 13% increase in total GSK3β from 23% before to 36% after the insulin treatment (Fig. 4f, Phos-tag, bottom panel). The levels of active Tyr216-phosphorylated GSK3β were consistently reduced about 10%. Thus, the increase in the phospho-Ser9 levels agreed roughly with the decrease in the levels of Tyr216-phosphorylated GSK3β on Phos-tag SDS-PAGE. However, this change was much less than those expected from the data of Ser9 phosphorylation. The active GSK3β still maintained its level high after insulin stimulation. Assuming that GSK3β with a single phosphorylation at Tyr216 is completely active in cells, the GSK3β activity would be more precisely estimated using the phospho-Tyr216 levels than the phospho-Ser9 levels. Taken together, it may be concluded that the insulin treatment does not inhibit GSK3β activity so much as those had been thought.

Nevertheless, there have been many reports describing a correlation, for example in the case of growth factor stimulation of cells, between the increase in the Ser9 phosphorylation of GSK3β and the possible outcomes resulting from the inhibition of GSK3β^45, 46^. This apparent disagreement may be explained by one of the following scenarios. One scenario is that GSK3β is not involved in those cellular activities, at least, as downstream of insulin stimulation. Second is that only a fraction of GSK3β is functionally active, and such GSK3β is inhibited by Ser9 phosphorylation upon cell stimulation. In fact, there is a report that shows the specific cellular localization of pS9 GSK3β only at growth cone of cultured neurons^47^. If this is the case, Ser9 phosphorylation can still be used as the indicator of GSK3β activity, as previously used. If so, our results raise questions of where and how functional GSK3β is expressed and how the majority of phospho-Tyr216 GSK3β is kept in the inactive state. Third scenario is that phosphorylation at Ser9 and inhibition of GSK3β activity could occur in parallel and do not necessarily have a cause-and-result relationship^42^. If this is true, the activity would be regulated by other factors, such as cellular localization or interaction with binding proteins^43^. Following Wnt signaling, it is known that GSK3β activity is suppressed independently of Ser9 phosphorylation^48, 49^. GSK3β forms multiprotein complexes and would be regulated by interactions with other proteins in the complexes. If so, the amount of phospho-Tyr216 GSK3β may not simply reflect the levels of functional, active GSK3β. In any case, it would be important to know the levels of each phosphoisotype of GSK3β in cells. The Phos-tag method will be a powerful tool for further investigations of the functional aspects of GSK3β phosphoisotypes.

## Methods

### Mutant GSK3β construct

A GSK3β-HA expression plasmid was provided by R. S. Jope at the University of Miami. The Ala mutant of GSK3β at Ser9 (S9A), Phe mutant at Tyr216 (Y216F), and double mutant at Ser9/Tyr216 (AF) were generated by site-directed mutagenesis using mouse GSK3β as a template. The primers used in this study were: 5′-CGGCCCAGAACCACCGCCTTGCGGAGAGC-3′ and 5′-GCTCTCCGCAAAGGCGGTGGTTCTGGGCCG–3′ for S9A, and 5′-GAACCCAATGTTTCGTTTATCTGTTCTCGG-3′ and 5′-CCGAGAACAATAAACGAAACATTGGGTTC-3′ for Y216F. The mutations were confirmed by DNA sequencing.

### Preparation of brain extracts

All animal experiments were performed according to the guidelines for animal experimentation of Tokyo Metropolitan University (in accordance with the Society for Neuroscience Guidelines). The study was approved by the Research Ethics Committee of Tokyo Metropolitan University (approval numbers; A28-10 and A27-4). All efforts were made to reduce the sufferings of animals used. ICR mice at different age groups were obtained from Sankyo Laboratory Service (Tokyo, Japan). The mouse was housed in a temperature-controlled room under 12 h light/dark cycle with free access to food and water. Mouse brains were dissected into cerebral cortices, cerebella, olfactory bulbs and hippocampi immediately after decapitation. The brain regions were homogenized using a Teflon-pestle homogenizer in 10 vol (v/w) of 20 mM HEPES, pH 7.4, 1 mM EGTA, 100 mM NaCl, 0.1% Nonidet P-40, 2 mM MgCl_2_, 10 mM NaF, 10 mM β-glycerophosphate, 10 μg/ml Pefabloc, 10 μg/ml leupeptin and 1 mM dithiothreitol. After centrifugation at 18,000 × g for 20 min, the supernatant was used as the brain extract. db/db mice, a model of Type 2 diabetes, and age matched non-diabetic wild type (WT) mice were obtained from CREA Japan, Inc. (Tokyo, Japan). Cerebral cortices were dissected from an 11-week-old male mouse and the brain extract was prepared as described above for the ICR mice.

### Cell and neuron culture

CHO-K1, HEK293, COS-7, SH-SY5Y and PC-12 cells were maintained in Dulbecco’s Modified Eagle’s Medium (DMEM, Sigma-Aldrich, St. Louis, MO) supplemented with 10% fetal bovine serum, 100 U/ml penicillin and 0.1 mg/ml Streptomycin. CHO-K1 cells were transfected with a plasmid encoding GSK3β WT or the S9A, Y216F or AF mutant using Lipofectamine 2000 (Thermo Fisher Scientific, Waltham, MA), or HilyMax (Dojindo Laboratories, Kumamoto, Japan) according to the manufacturer’s instruction. All transfections were performed in triplicate. To study the effect of GSK3β inhibitors, CHO-K1 cells were plated at a density of 1 × 10^5^ cells/ml and treated with SB415286 (Sigma-Aldrich, St. Louis, MO) at 20 μg/ml or LiCl at 10 mM for 5 and 10 h. For insulin study, the culture medium was replaced with serum-free media for 6 h, and then insulin (Eli Lilly, Tokyo, Japan) was added at 50 µU/ml to the medium for 10, 30 and 60 min.

Primary neurons were prepared from the mouse brain cortex at embryonic day 16 (E16) and plated on polyethyleneimine-coated dishes in DMEM and Ham’s F-12 (1:1) supplemented with 5% fetal bovine serum, 5% horse serum, 100 U/ml penicillin and 0.1 mg/ml streptomycin at a density of 1.5 × 10^6^ cells/ml^50^. The medium was changed to Neurobasal medium supplemented with 2% B-27 (Invitrogen, Grand Island, NY), 0.5 mM L-glutamine, 100 U/ml penicillin and 0.1 mg/ml streptomycin after 4 h of plating. Half of the medium was replaced with fresh medium every third day. For Insulin-like growth factor (IGF) or insulin treatment, primary neurons at 5 DIV were cultured in the absence of B-27 for 12 h, and then IGF-1 (Astellas, Tokyo, Japan) and insulin were added at 100 ng/ml and 50 μU/ml respectively, in the medium for 10, 30 and 60 min, followed by cell collection.

### Immunoprecipitation of GSK3β and kinase assay

CHO-K1 cells expressing GSK3β-HA or its mutants and HEK293 cells treated with insulin were lysed in 20 mM Hepes, pH 7.4, 0.5 mM EGTA, 0.1 mM EDTA, 0.15 M NaCl, 1 mM MgCl_2_, 10 μg/ml leupeptin, 0.4 mM Pefabloc and 0.5% (v/v) Nonidet P-40. The lysate was centrifuged at 18,000 x g for 15 min, the supernatant was collected and incubated with 200 μg/ml anti-HA (Y-11) (#sc-805, Santa Cruz Biotechnology) for transfected GSK3β-HA or its mutants and 100 μg/ml anti-GSK3β (#9315, Cell Signaling Technologies) for endogenous GSK3β along with 5 μl {50% (v/v) slurry} of Dynabeads Protein G (Thermo Fisher, CA) by rotation for the respective experiments. Beads were washed in the same buffer. The kinase activity of the immunoprecipitates was measured using a GSK3β peptide (pA3b-GS-1, 1 mg/ml) and [γ-^32^P]ATP as substrates in kinase buffer (10 mM MOPS, pH 6.8, 5 mM MgCl_2_, 0.1 mM EGTA, 0.1 mM EDTA) for 5 min by Cerenkov counting in a liquid scintillation counter (LS6500, Beckman Coulter, CA). The amino acid sequence of the GSK3β peptide is YRRAAVPPAPSLSRHSSPHQSpEDEE (singer letter representation), in which the prime phosphorylation site is Ser at fifth amino acid from the C-terminus^20, 51^.

### Dephosphorylation of GSK3β

CHO-K1 cells expressing GSK3β were lysed in 50 mM Tris HCl, pH 8.0, 1 mM MgCl_2_, 100 mM NaCl, 0.5% Nonidet P-40, 0.4 mM Pefabloc, 10 μg/ml leupeptin and 1 mM dithiothreitol on ice for 5 min. After centrifugation at 18,000 × g for 15 min, the supernatant was collected as the cell extract. The extract was incubated with 2.5 U/ml *Escherichia coli*. alkaline phosphatase (Sigma-Aldrich, St. Louis, MO) in 50 mM Tris, pH 8.8, 0.15 M NaCl, 0.1 mM EDTA, 0.1 mM EGTA and 1 mM MgCl_2_ at 37 °C for 3 h.

### Laemmli’s SDS-PAGE, Phos-tag SDS-PAGE and immunoblotting

The cells were washed with 1x PBS and lysed in 20 mM HEPES, pH 7.4, 1 mM MgCl_2_, 100 mM NaCl, 0.5% Nonidet P-40, 0.4 mM Pefabloc, 10 μg/ml leupeptin, 10 mM Sodium Fluoride, 10 mM β-Glycerophosphate and 1 mM dithiothreitol on ice for 5 min^52^. After centrifugation at 18,000 x g for 15 min, the supernatant was collected and the protein concentration was measured using Coomassie Protein Assay reagent (Thermo Scientific, IL) followed by denaturation. Laemmli’s SDS-PAGE was performed using 10% (w/v) polyacrylamide gels. Proteins were transferred to PVDF membranes (Millipore, Bedford, MA) using a semidry blotting apparatus. The membranes were probed with antibodies against GSK3β (9315, Cell Signaling Technologies, 1:1000), p-S9 GSK3β (9336, Cell Signaling Technologies, 1:750), p-Y216 GSK3β (612312, BD Transduction Laboratories, 1:1000), Actin (A2066, Sigma-Aldrich, 1:2000), Akt (9272, Cell Signaling Technologies, 1:500), p-Akt (9271, Cell Signaling Technologies, 1:500, β-Catenin) (610154, BD Transduction Laboratories, 1:1000), p-β-Catenin (9561, Cell Signaling Technologies, 1:200), GSK3α (ab40870, abcam, 1:5000). The appropriate horseradish peroxidase-conjugated secondary antibodies (goat anti-mouse IgG, P0447, Dako, 1:1000; swine anti-rabbit IgG, P0399, Dako, 1:1000) were then applied. The immunoreactions were developed with the Immobilon Western Chemiluminescent HRP substrate (Millipore, Billerica, MA) and the immunoreactions were captured into a FUSION SL apparatus (Vilber Lourmat, Wembley, Australia) using an automated ‘paste marker’ function.

Phosphorylation states of GSK3β were examined using Phos-tag SDS-PAGE, which is a phospho-affinity SDS-PAGE developed by Kinoshita *et al*.^18^. Phos-tag acrylamide was purchased from Wako Chemicals (Osaka, Japan). Separating gels were made by copolymerization of acrylamide with Phos-tag acrylamide. The migration of phosphoproteins in the separating gel are less than those of their nonphosphorylated counterpart because the Phos-tag traps phosphoproteins reversibly during electrophoresis (see Kinoshita *et al*., Nat Protocol., 2009 for a ref. 53). Phos-tag SDS-PAGE was performed on 7.5% polyacrylamide gels containing 50 μM Phos-tag acrylamide and 100 μM MnCl_2_^18, 19, 54^ in 10 mA current. This makes the separation of proteins possible by detecting shifts in the mobility of phosphoproteins and identified as various bands based on the degree of phosphorylation. The separated proteins were transferred to PVDF membranes using a submerged blotting apparatus. Immunoreaction of the membrane is carried out as described above.

### Quantification and statistical analysis

The band intensities and banding profiles were analysed with Image J software. The data are represented as mean ± s.e.m. from more than three independent set of experiments. Statistical significance was determined by Student’s *t*-test or one–way ANOVA using GraphPad PRISM software version 5.0. P < 0.05 was considered significantly different.

## Electronic supplementary material

Supplementary Information

## References

[1] Fiol CJ, Mahrenholz AM, Wang Y, Roeske RW, Roach PJ (1987). Formation of protein kinase recognition sites by covalent modification of the substrate. Molecular mechanism for the synergistic action of casein kinase II and glycogen synthase kinase 3. J. Biol. Chem..

[2] Frame S, Cohen P (2001). GSK3 takes centre stage more than 20 years after its discovery. Biochem. J..

[3] Doble BW, Woodgett JR (2003). GSK-3: tricks of the trade for a multi-tasking kinase. J. Cell Sci..

[4] Jope RS, Johnson GV (2004). The glamour and gloom of glycogen synthase kinase-3. Trends Biochem. Sci..

[5] Takashima A (2006). GSK-3 is essential in the pathogenesis of Alzheimer’s disease. J. Alzheimers Dis..

[6] Hernandez F, Lucas JJ, Avila J (2013). GSK3 and tau: two convergence points in Alzheimer’s disease. J. Alzheimers Dis..

[7] Lee J, Kim MS (2007). The role of GSK3 in glucose homeostasis and the development of insulin resistance. Diabetes Res. Clin. Pract..

[8] Hughes K, Nikolakaki E, Plyte SE, Totty NF, Woodgett JR (1993). Modulation of the glycogen synthase kinase-3 family by tyrosine phosphorylation. EMBO J..

[9] Lochhead PA (2006). Chaperone-dependent GSK3beta transitional intermediate mediates activation-loop autophosphorylation. Mol. Cell..

[10] Cole A, Frame S, Cohen P (2004). Further evidence that the tyrosine phosphorylation of glycogen synthase kinase-3 (GSK3) in mammalian cells is an autophosphorylation event. Biochem. J..

[11] Goc A (2014). Targeting Src-mediated Tyr216 phosphorylation and activation of GSK-3 in prostate cancer cells inhibit prostate cancer progression *in vitro* and *in vivo*. Oncotarget..

[12] Sutherland C, Leighton IA, Cohen P (1993). Inactivation of glycogen synthase kinase-3 beta by phosphorylation: new kinase connections in insulin and growth-factor signalling. Biochem. J..

[13] Jensen J, Brennesvik EO, Lai YC, Shepherd PR (2007). GSK-3beta regulation in skeletal muscles by adrenaline and insulin: evidence that PKA and PKB regulate different pools of GSK-3. Cell Signal..

[14] Dajani R (2001). Crystal structure of glycogen synthase kinase 3 beta: structural basis for phosphate-primed substrate specificity and autoinhibition. Cell..

[15] Ter Haar E (2001). Structure of GSK3 beta reveals a primed phosphorylation mechanism. Nat. Struct. Biol..

[16] Wu D, Pan W (2010). GSK3: a multifaceted kinase in Wnt signaling. Trends Biochem. Sci..

[17] Kaidanovich-Beilin O, Woodgett JR (2011). GSK-3: Functional Insights from Cell Biology and Animal Models. Front. Mo.l Neurosci..

[18] Kinoshita E, Kinoshita-Kikuta E, Takiyama K, Koike T (2006). Phosphate-binding tag, a new tool to visualize phosphorylated proteins. Mol. Cell Proteomics..

[19] Hosokawa T, Saito T, Asada A, Fukunaga K, Hisanaga S (2010). Quantitative measurement of *in vivo* phosphorylation states of Cdk5 activator p35 by Phos-tag SDS-PAGE. Mol. Cell Proteomics..

[20] Hoshi M (1996). Regulation of mitochondrial pyruvate dehydrogenase activity by tau protein kinase I/glycogen synthase kinase 3beta in brain. Proc Natl Acad Sci USA..

[21] Mukai F, Ishiguro K, Sano Y, Fujita SC (2002). Alternative splicing isoform of tau protein kinase I/glycogen synthase kinase 3beta. J. Neurochem..

[22] Jope RS (2003). Lithium and GSK-3: one inhibitor, two inhibitory actions, multiple outcomes. Trends Pharmacol. Sci..

[23] Coghlan MP (2000). Selective small molecule inhibitors of glycogen synthase kinase-3 modulate glycogen metabolism and gene transcription. Chem. Biol..

[24] Culbert AA (2001). GSK-3 inhibition by adenoviral FRAT1 overexpression is neuroprotective and induces Tau dephosphorylation and beta-catenin stabilisation without elevation of glycogen synthase activity. FEBS Lett..

[25] MacAulay K (2003). Use of lithium and SB-415286 to explore the role of glycogen synthase kinase-3 in the regulation of glucose transport and glycogen synthase. Eur. J. Biochem..

[26] Cross DA, Alessi DR, Cohen P, Andjelkovich M, Hemmings BA (1995). Inhibition of glycogen synthase kinase-3 by insulin mediated by protein kinase B. Nature..

[27] Summers SA (1999). The role of glycogen synthase kinase 3beta in insulin-stimulated glucose metabolism. J. Biol. Chem..

[28] Bhat RV, Budd Haeberlein SL, Avila J (2004). Glycogen synthase kinase 3: a drug target for CNS therapies. J. Neurochem..

[29] Liang MH, Chuang DM (2007). Regulation and function of glycogen synthase kinase-3 isoforms in neuronal survival. J. Biol. Chem..

[30] Singh B (2012). Resistance to trophic neurite outgrowth of sensory neurons exposed to insulin. J. Neurochem..

[31] Plattner F, Angelo M, Giese KP (2006). The roles of cyclin-dependent kinase 5 and glycogen synthase kinase 3 in tau hyperphosphorylation. J. Biol. Chem..

[32] de la Monte SM, Wands JR (2008). Alzheimer’s disease is Type 3 diabetes - Evidence reviewed. J. Diabetes Sci. Technol..

[33] Clodfelder-Miller B, De Sarno P, Zmijewska AA, Song L, Jope RS (2005). Physiological and pathological changes in glucose regulate brain Akt and glycogen synthase kinase-3. J. Biol. Chem..

[34] Jolivalt CG (2008). Defective insulin signaling pathway and increased glycogen synthase kinase-3 activity in the brain of diabetic mice: parallels with Alzheimer’s disease and correction by insulin. J. Neurosci. Res..

[35] El Khoury NB (2016). Hypothermia mediates age-dependent increase of tau phosphorylation in db/db mice. Neurobiol. Dis..

[36] Grabinski T, Kanaan NM (2016). Novel Non-phosphorylated Serine 9/21 GSK3B/A Antibodies: Expanding the Tools for Studying GSK3 Regulation. . Front Mol Neurosci..

[37] Ding Q (2005). Erk associates with and primes GSK-3beta for its inactivation resulting in upregulation of beta-catenin. Mol. Cell..

[38] Thornton TM (2008). Phosphorylation by p38 MAPK as an alternative pathway for GSK3beta inactivation. Science..

[39] Ryves WJ, Harwood AJ (2001). Lithium inhibits glycogen synthase kinase-3 by competition for magnesium. Biochem. Biophys. Res. Commun..

[40] De Sarno P, Li X, Jope RS (2002). Regulation of Akt and glycogen synthase kinase-3 beta phosphorylation by sodium valproate and lithium. Neuropharmacology..

[41] Forde JE, Dale TC (2007). Glycogen synthase kinase 3: a key regulator of cellular fate. Cell Mol. Life Sci..

[42] Beurel E, Grieco SF, Jope RS (2015). Glycogen synthase kinase-3 (GSK3): regulation, actions, and diseases. Pharmacol. Ther..

[43] Hur EM, Zhou FQ (2010). GSK3 signalling in neural development. Nat. Rev. Neurosci..

[44] Garcia-Segura LM, Diz-Chaves Y, Perez-Martin M, Estradiol DM (2007). Insulin-like growth factor-I and brain aging. Psychoneuroendocrinology..

[45] MacDonald BT, Tamai K, He X (2009). Wnt/beta-catenin signaling: components, mechanisms, and diseases. Dev. Cell..

[46] Valvezan AJ, Klein PS (2012). GSK-3 and Wnt Signaling in Neurogenesis and Bipolar Disorder. Front. Mol. Neurosci..

[47] Mukai J (2015). Molecular Substrates of Altered Axonal Growth and Brain Connectivity in a Mouse Model of Schizophrenia. Neuron.

[48] Blankesteijn WM, van de Schans VA, ter Horst P, Smits JF (2008). The Wnt/frizzled/GSK-3 beta pathway: a novel therapeutic target for cardiac hypertrophy. Trends Pharmacol. Sci..

[49] Ng SS (2009). Phosphatidylinositol 3-kinase signaling does not activate the wnt cascade. J. Biol. Chem..

[50] Saito T (2003). Developmental regulation of the proteolysis of the p35 cyclin-dependent kinase 5 activator by phosphorylation. J. Neurosci..

[51] Woodgett JR (1989). Use of peptide substrates for affinity purification of protein-serine kinases. Anal Biochem..

[52] Kimura T (2013). Isomerase Pin1 stimulates dephosphorylation of tau protein at cyclin-dependent kinase (Cdk5)-dependent Alzheimer phosphorylation sites. J. Biol. Chem..

[53] Kinoshita E, Kinoshita-Kikuta E, Koike T (2009). Separation and detection of large phosphoproteins using Phos-tag SDS-PAGE. Nat Protoc..

[54] Kimura T (2016). The Abundance of Nonphosphorylated Tau in Mouse and Human Tauopathy Brains Revealed by the Use of Phos-Tag Method. Am. J. Pathol..

