# Supplementary material for: In vivo regulation of glycogen synthase kinase 3β activity in neurons and brains
Source: Sci Rep. 2017 Aug 17;7:8602. doi: 10.1038/s41598-017-09239-5 (PMC5561119; doi:10.1038/s41598-017-09239-5)

**Supplementary Figures**

***In vivo* regulation of glycogen synthase kinase 3β** **activity in neurons and brains**

**Ambika Krishnankutty^1^, Taeko Kimura^1^, Taro Saito^1^, Kyota Aoyagi^2^, Akiko Asada^1^, Shin-Ichiro Takahashi^3^, Kanae Ando^1^, Mica Ohara-Imaizumi^2^, Koichi Ishiguro^4^, Shin-ichi Hisanaga^1,*^**

^1^Laboratory of Molecular Neuroscience, Department of Biological Sciences, Graduate School of Science, Tokyo Metropolitan University, Hachioji, Tokyo, ^2^Department of Biochemistry, Kyorin University School of Medicine, Mitaka, Tokyo, ^3^Department of Animal Sciences, Graduate School of Agriculture and Life Sciences, The University of Tokyo, Bunkyo, Tokyo and ^4^Department of Neurology, Graduate School of Medicine, Juntendo University, Bunkyo, Tokyo, Japan


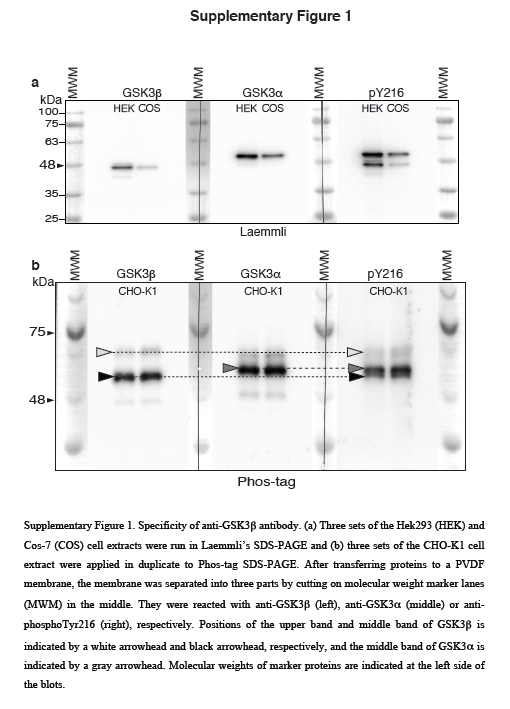


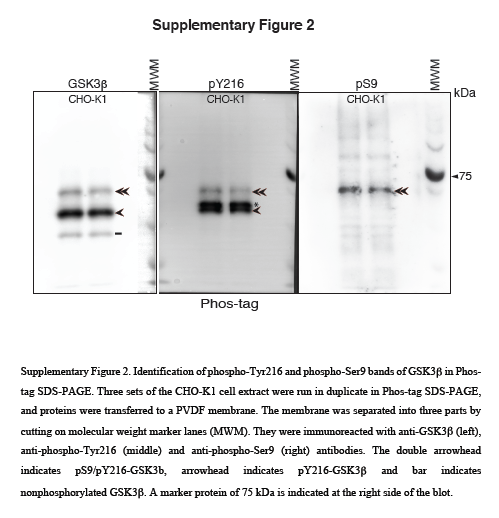


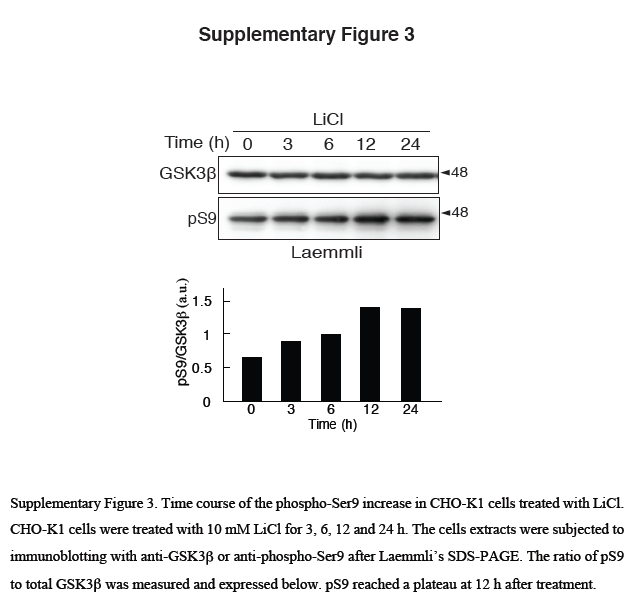


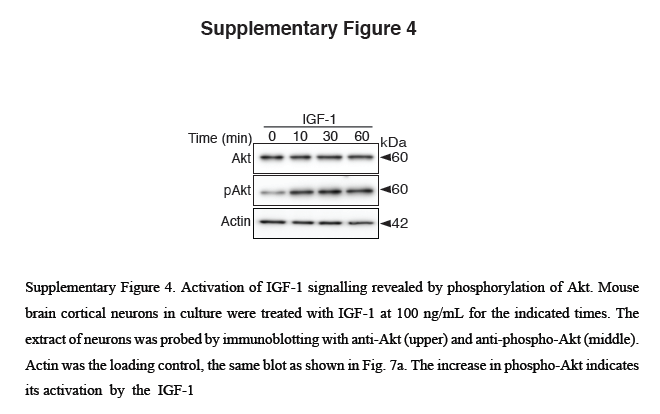


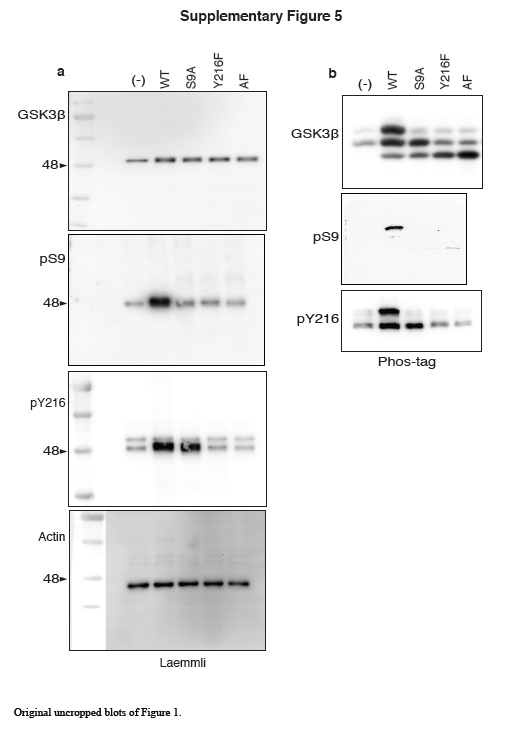


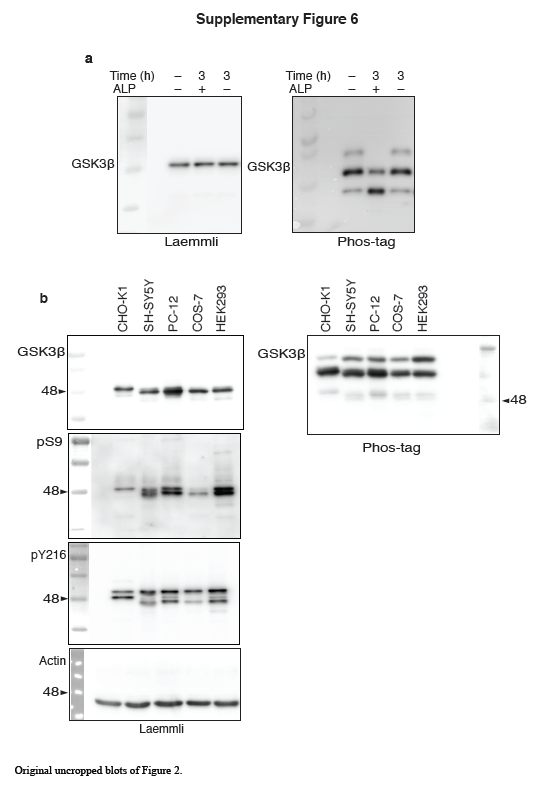


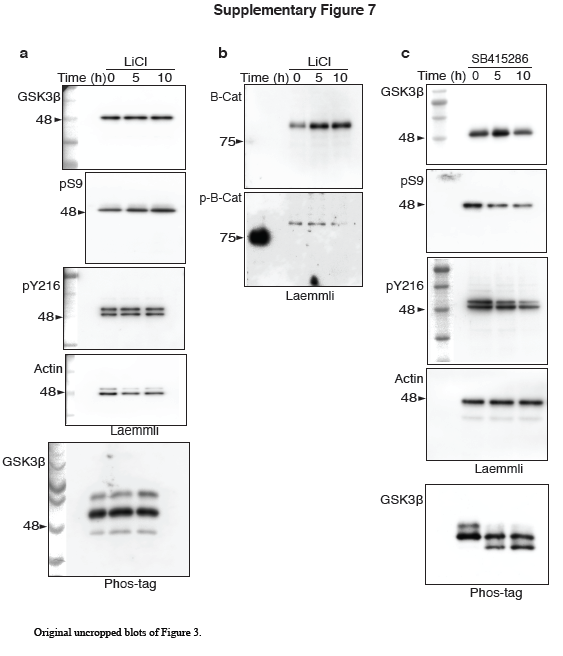


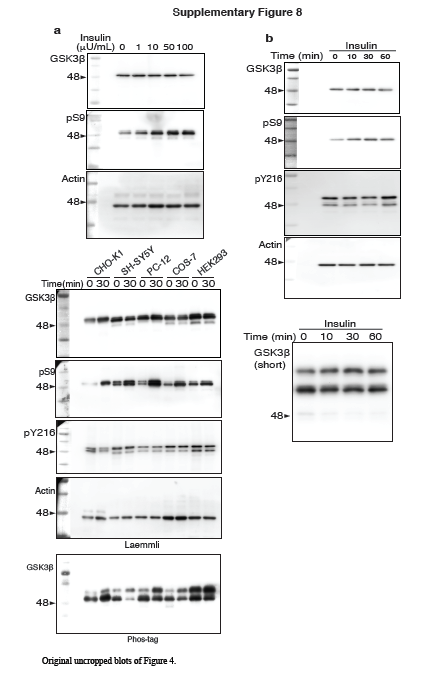


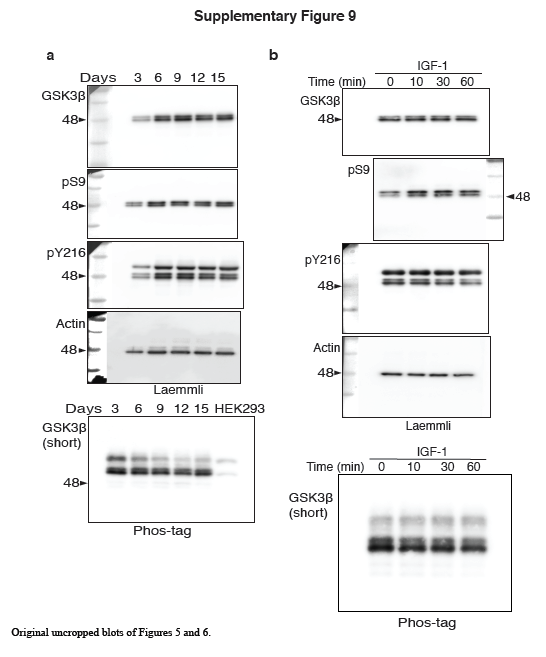


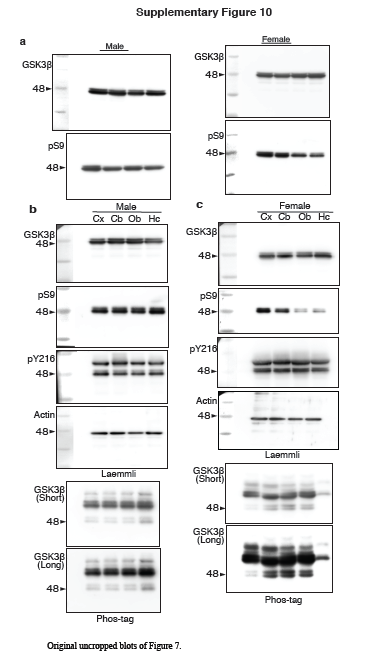


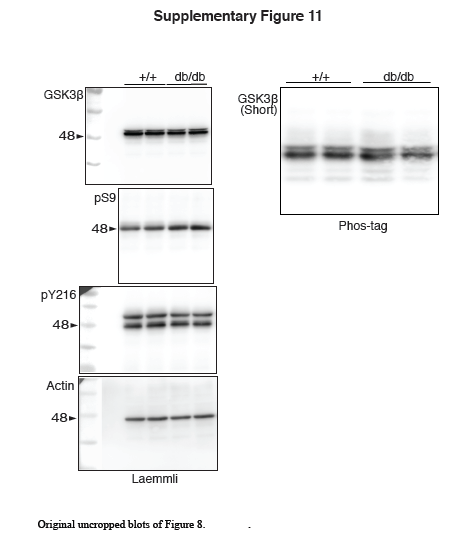

Supplement: Supplementary file 1 — Supplementary Information [file 41598_2017_9239_MOESM1_ESM.doc]
